# The Effect of GGBFS, Quartz Flour, Micro-Calcite, and Their Combinations on the Structure and Properties of Polymer Concrete

**DOI:** 10.3390/polym18202462

**Published:** 2026-10-09

**Authors:** Yasin Onuralp Özkılıç, Evgenii M. Shcherban’, Sergey A. Stel’makh, Diana M. Shakhalieva, Andrei Chernil’nik, Natalya Shcherban’, Li Li, Dmitry R. Mailyan, Shengwen Tang, Alexey N. Beskopylny

**Affiliations:** 1 Department of Unique Buildings and Constructions Engineering, Don State Technical University, 344003 Rostov-on-Don, Russia; sstelmah@donstu.ru (S.A.S.); achernilnik@donstu.ru (A.C.); ndocenko@donstu.ru (N.S.); 2 Department of Civil Engineering, Faculty of Engineering, Necmettin Erbakan University, Konya 42000, Turkey; 3 Department of Technical Sciences, Western Caspian University, Baku 1001, Azerbaijan; 4 Department of Engineering Geometry and Computer Graphics, Don State Technical University, 344003 Rostov-on-Don, Russia; etsherban@donstu.ru; 5 Department of Design, Don State Technical University, 344003 Rostov-on-Don, Russia; delshaeva@donstu.ru; 6 College of Water Resources and Architectural Engineering, Northwest A&F University, Yangling 712100, China; drlili@nwafu.edu.cn; 7 Department of Reinforced Concrete Structures, Faculty of Industrial and Civil Engineering, Don State Technical University, Gagarin, 1, 344003 Rostov-on-Don, Russia; dmailyan@donstu.ru; 8 State Key Laboratory of Water Resources and Hydropower Engineering Science, Wuhan University, Wuhan 430072, China; tangsw@whu.edu.cn; 9 Department of Transport Systems, Faculty of Roads and Transport Systems, Don State Technical University, 344003 Rostov-on-Don, Russia

**Keywords:** polymer concrete, mineral fillers, compressive strength, polymer matrix, polymer concrete microstructure

## Abstract

This research aims to develop new types of polymer concrete composites. The main objective of the work was to investigate the influence of various types of mineral fillers (ground granulated blast-furnace slag (GGBFS), quartz flour (QF), micro-calcite (MC)) and their combinations on the properties of polymer concrete (PC) and to develop new effective PC compositions. An optimal PC composition was developed and seven series of experimental PC samples with different types of mineral fillers were manufactured: 1PC (GGBFS); 2PC (QF); 3PC (MC); 4PC (GGBFS + QF); 5PC (GGBFS + MC); 6PC (QF + MC); 7PC (GGBFS + QF + MC). The following parameters were measured: density, compressive strength, water absorption, and thermal conductivity. Scanning electron microscopy was used to assess the PC microstructure. To quantitatively evaluate the significance and influence of the mineral filler type on the PC properties, an analysis of variance was performed. The experimental results showed that the mineral fillers GGBFS, QF and MC and their combinations affect the properties of PC, especially its strength. The composition of 5PC turned out to be optimal: epoxy resin 15%; hardener 1.5%; plasticizer 1.5%; quartz sand 30%; crushed granite 30%; GGBFS 11%; MC 11%. Polymer concrete of composition 5PC had a density of 2263 kg/m^3^, a maximum compressive strength of 103.8 MPa, a minimum water absorption of 0.25% and a thermal conductivity coefficient of 0.466 W/m·K. The PC of all compositions had similar morphology, with a uniform structure and a randomly distributed pore size ranging from 81.27 µm to 384.9 µm. Microcracks were predominantly concentrated at the phase boundaries and propagated through the polymer matrix, enveloping the filler grains. The study yielded effective composite materials that could potentially be used in mechanical engineering as well as in industrial and civil construction, following durability testing and the determination of the full range of required properties.

## 1. Introduction

Currently, polymeric materials for various purposes are actively used in industrial and civil construction [1,2,3]. Polymer materials have high corrosion and chemical resistance, which makes their field of application quite wide [4,5,6]. Polymeric materials gain unique properties for practical use by applying diverse formulation and technological methods [7,8]. For example, modification of recycled high-density polyethylene with 1.5% nanoclay particles allows obtaining environmentally friendly composites for use in containers, construction, and machinery, with a tensile strength of 31.27 MPa and an elastic modulus of 2.39 GPa [9]. Plasma treatment of textile polymeric materials used in the technology of building enclosing structures ensures an increase in their surface roughness, improves coating adhesion, and improves their hydrophobic properties [10]. Reinforcement of asphalt concrete pavements with carbon geogrids increases their dynamic stability and flexural strength by 12.80–13.74% and 4.53–37.47%, respectively [11]. The polypropylene composite’s strength and elastic modulus are improved by incorporating carbon nanofibers and wollastonite [12]. Mixing plant fillers (birch wood flour, hemp shives, hemp fiber) with poly(3-hydroxybutyrate) and molding samples by hot pressing allows us to obtain effective biodegradable composites for industry [13]. Modification of polymer composites with carbon nanotubes increases thermal conductivity by 168% and mechanical strength by 62.72% [14]. Recycled polyethylene in combination with wood fibers has optimal performance properties for use as a structural material [15]. The combination of polylactic acid with crushed volcanic rock, palm waste, and quartz sand in optimal dosages produced a composite with enhanced thermal insulation and strength properties [16]. Reinforcement of reinforced concrete beams with silicate-modified epoxy resin plates increases their load-bearing capacity and reduces ductility [17]. Epoxy resin, when used as an internal concrete additive, hinders aggressive ion penetration and reduces sulfate corrosion damage, all while preserving concrete’s performance [18]. A study on the properties of composites containing nylon granules under high-temperature exposure revealed that, at 600 °C, compressive strength and the modulus of elasticity decrease by approximately 59% and 75%, respectively [19]. Treating recycled concrete aggregate with liquid polyvinyl alcohol increases its density and reduces water absorption [20]. Using polymer resins, polymer reinforcement, and polymer fibers in cement concrete technologies in optimal quantities allows for targeted modification and improvement of their properties [21,22,23]. The study [24] examines a mechanistic approach to restoring severely aged asphalt using rejuvenators and demonstrates their effectiveness. The epoxy groups of the epoxy-sulfonated oligomer react with carboxylic acids in the aged asphalt to form ester bonds, facilitating chemical integration between the epoxy-sulfonated oligomer and the components of the aged asphalt.

Among the wide range of polymeric materials, the improvement of new types of polymer concrete with high mechanical characteristics and unique performance properties appears particularly promising [25,26]. Polymer concrete is produced by mixing the polymer with mineral fillers and aggregates [27,28]. The polymer acts as a binder, combining all the raw materials into a composite that hardens quickly and exhibits high strength properties, is resistant to freeze–thaw cycles, and has low permeability to water and other various aggressive chemical environments [29,30]. When developing polymer concrete mixture recipes, it is important to consider aspects related to selecting the optimal ratio between the polymer binder and filler, and the selection of compatible mineral components with optimal particle sizes, which ensure the densest possible packing of particles in the polymer matrix [31]. A prior development involved an epoxy resin-based polymer concrete, enhanced with functionalized carbon nanotubes, exhibiting a compressive strength of 78 MPa [32]. The mechanical properties of both polymer concrete and lightweight polymer concrete are significantly influenced by the ratio of resin and the amount of filler. The optimal resin content in polymer concrete was 20% by weight and, in lightweight polymer concrete, 45% by weight [33].

An investigation into the mechanical properties of epoxy-polymer concrete and its failure criteria under triaxial compression has shown that, at low levels of compression, the failure mode of the polymer concrete changes from longitudinal failure to inclined shear failure. At higher levels of compression, however, polymer concrete shows extension under compression [34]. Reinforcement of the epoxy matrix with tin slag and glass fiber allows obtaining a specimen with a compressive strength of 148.19 MPa [35]. In [36], polymer concretes were developed using waste aggregates from demolition and construction materials. These composites had high strength, and the epoxy resin content varied from 20% to 30%. Composites of polyester, sand, and waste recycled polyethylene and fiberglass demonstrate efficient recycling of waste materials, allowing the production of durable, environmentally friendly polymer composites with a strength of 56 MPa [37]. Precast columns filled with polymer concrete demonstrated greater performance than similar columns filled with high-strength concrete [38]. Polymer concrete based on vinyl ester resin and 5% natural perlite demonstrated excellent resistance to both high temperatures and freeze–thaw cycles in comparison with polymer concrete based on polyester resin [39]. Due to its superior damping, minimal thermal expansion, and adaptable structure, polymer concrete is considered a viable substitute for cast iron in creating precise machine frames. Compared to cast iron, polymer concrete has a damping coefficient several times higher, which contributes to increased machining accuracy and extends the service life of cutting tools [40]. In a study [41], the authors examined the impact of aggregate particle size on the mechanical properties of PC. According to the experimental findings, achieving optimal aggregate particle size distribution and a content of up to 83% within the composite enhances mechanical properties and decreases porosity. An epoxy-diane resin-based polymer composite for precision machine guides, featuring a compressive strength reaching 92.3 MPa, can be produced by optimizing the combination of fillers such as quartz sand, basalt, silicon carbide, and fiberglass [42]. The compressive strength reaches 78–85 MPa, with a damping coefficient of about 0.022 [43], when 22–26% epoxy resin is incorporated with 55–70% fine aggregate derived from granite quarry waste. The flexural strength of polymer concretes for high-precision machine tools is improved by up to 35% through the integration of prestressed carbon fibers, which also enhances load-bearing capacity and fracture resistance [44].

Optimizing the polymer concrete composition and selecting the best raw components are crucial for creating a highly efficient material for precision machine tool frames [45,46]. As the review indicates, various types of coarse aggregates (heavy, lightweight porous, and recycled demolition waste aggregates) and fine aggregates/fillers (quartz sand, perlite, tin slag) are used to develop optimal polymer concrete mix designs. However, there is a lack of research regarding the optimization of polymer concrete compositions using different types of mineral fillers. Ground granulated blast-furnace slag, micro-calcite, and quartz flour are the most popular and readily available mineral additives, making their use in polymer concrete production highly rational. Modifying the polymer matrix with mineral filler is expected to yield a comprehensive improvement in the composite’s performance properties. This research examines how different mineral fillers (slag, quartz flour, micro-calcite) and their mixtures impact the compressive strength, durability, density, water absorption, and thermal conductivity of PC, aiming to advance the understanding of synergistic design and optimization in this field. The scientific innovation is found in creating novel polymer concretes enhanced by various mineral fillers such as GGBFS, quartz flour, and micro-calcite, along with their mixtures. A key aspect is establishing the dependencies of the principal properties of polymer concretes on the modification parameters. The study’s objective is to explore the effects of diverse mineral fillers (GGBFS, quartz flour, micro-calcite) and their blends on polymer concrete characteristics and to devise new, effective compositions.

The goals of this research are:

– Assessing the properties of the main raw materials;
– Selecting optimal polymer concrete formulations and producing experimental samples;
– Determining the density, compressive strength, water absorption, and thermal conductivity of PC;
– Studying the structural features of modified polymer concrete matrices using SEM and EDS.

## 2. Materials and Methods

### 2.1. Materials

Experimental polymer concrete (PC) samples were prepared using raw materials based on epoxy resin.

1. ED-20 (ER) epoxy resin. Properties: epoxy group content—22.0%; hydroxyl group content—1.7%; dynamic viscosity—14 Pa × s; density—1.15 g/cm^3^. In polymer concrete, epoxy resin acts as a binding agent, uniting the mineral components into a single composite.
2. Polyethylenepolyamine (HP) hardener PEPA (EDGUS, Moscow, Russia). Properties: density—1.011 g/cm^3^; curing capacity—1.5 h; water content—2.0%; acid-titrated nitrogen content—19.5%. The curing agent plays a crucial role in the composition of polymer concrete, initiating the curing processes of the epoxy resin.
3. DEG-1 (P) plasticizer (Polimax, St. Petersburg, Russia). Characteristics: epoxy group content—25.3%; saponifiable chlorine content—1.45%; dynamic viscosity at 25 °C—0.07 Pa × s. A plasticizer is added to increase the plasticity and elasticity of the polymer matrix.
4. Quartz sand (QS). Characteristics: bulk density—1375 kg/m^3^; dust and clay particle content—0.11%.
5. Granite crushed stone (CrS) (Granite, Kamennogorskoye, Russia). Properties: bulk density—1458 kg/m^3^; resistance to fragmentation—11.2 wt. %; the content of lamellar and acicular grains—3.1 wt. %, grain size 5–10 mm.

**Figure 1:**
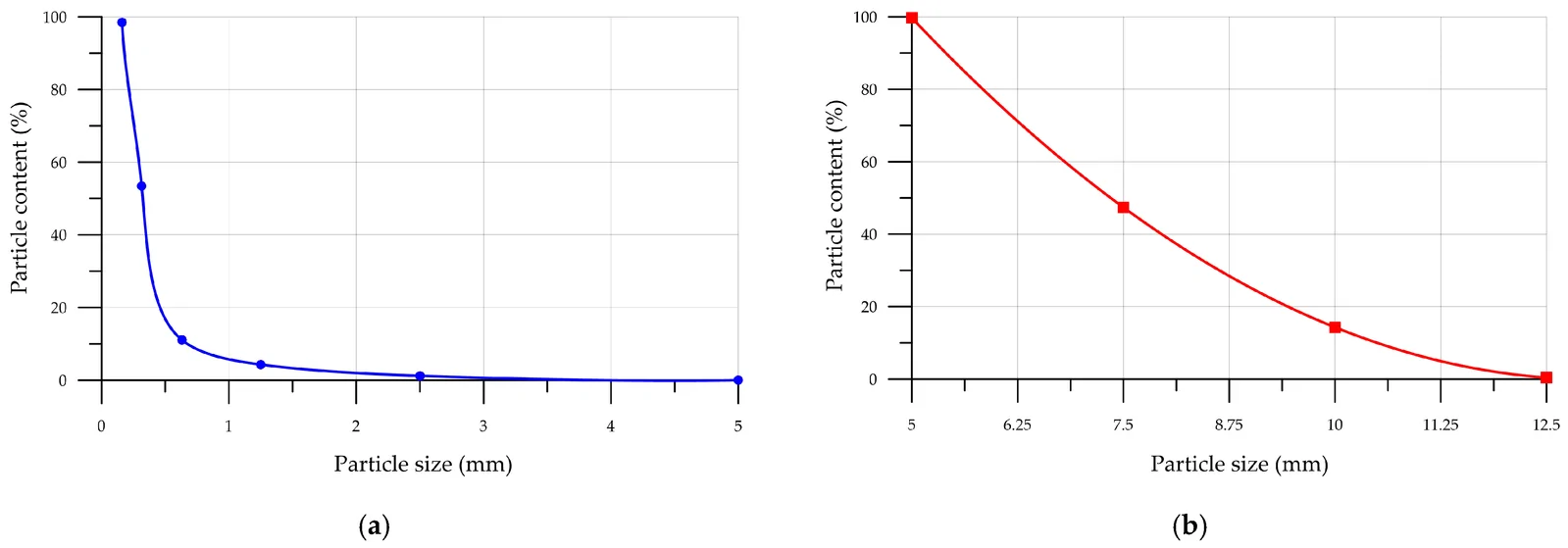
Particle size distribution curves: (**a**) QS; (**b**) CrS.

Figure 1 illustrates the particle size distribution curves for QS and CrS.

The cumulative percentage of material retained on sieves 2.5, 1.25, 0.63, 0.315, and 0.16 for QS amounts to 1.2%, 4.3%, 11.1%, 53.4%, and 98.5% (Figure 1a). The fineness modulus of QS is 1.69. CrS has grain sizes of 5–10 mm.

**Figure 2:**
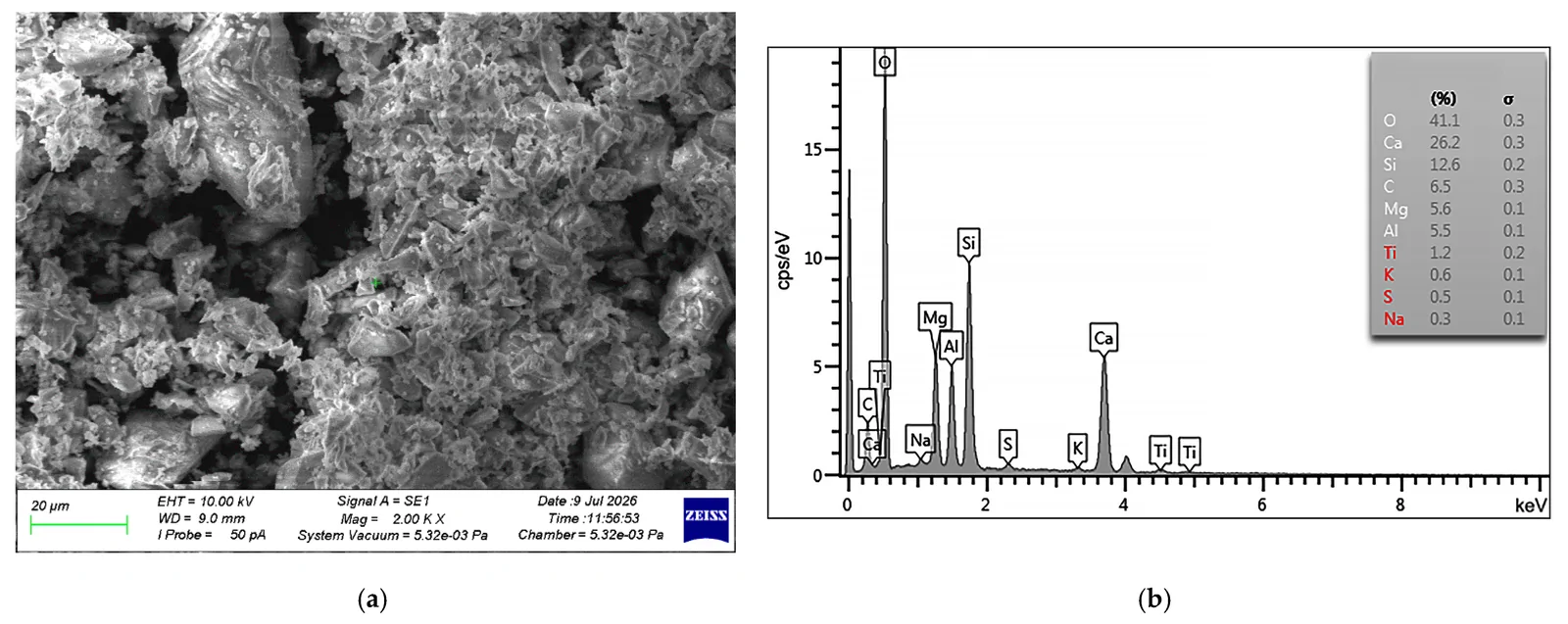
GGBFS: (**a**) SEM image at 2000× magnification; (**b**) EDS spectrum.

**Figure 3:**
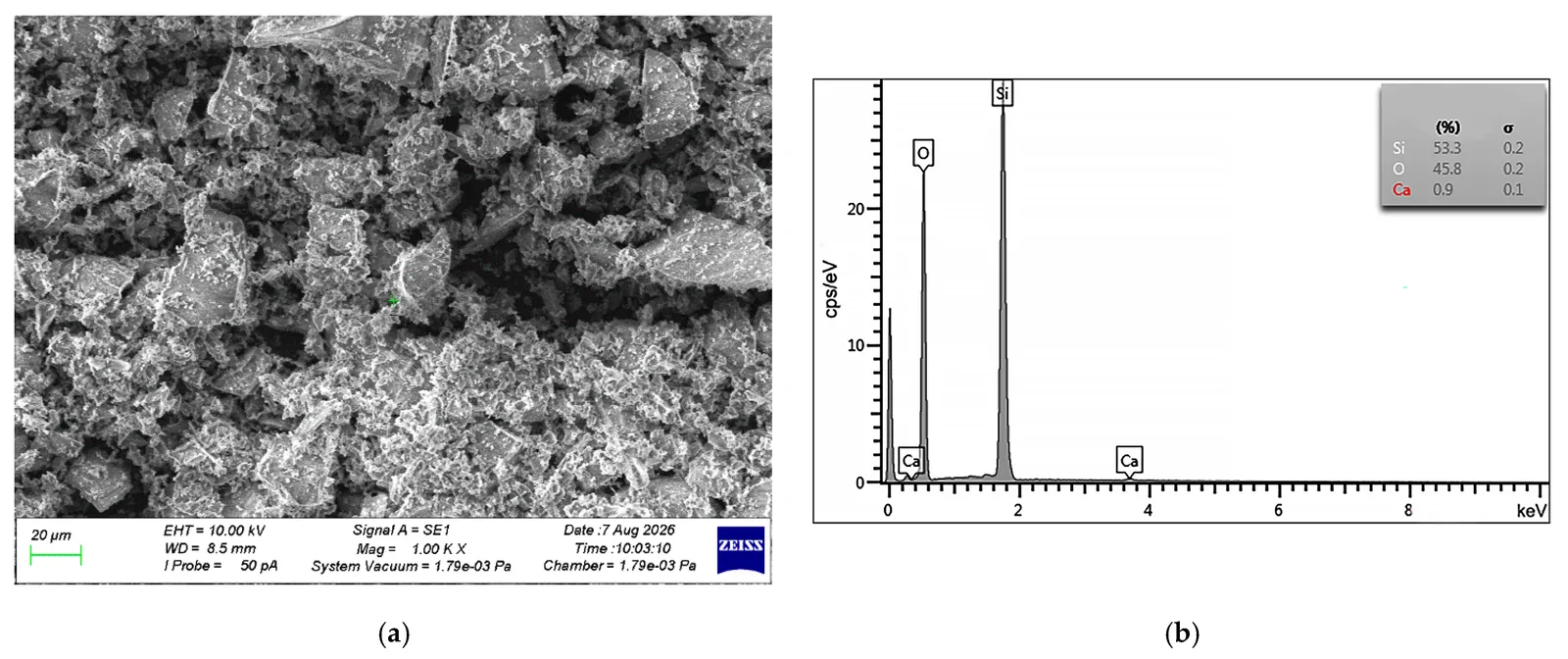
QF: (**a**) SEM image at 1000× magnification; (**b**) EDS spectrum.

**Figure 4:**
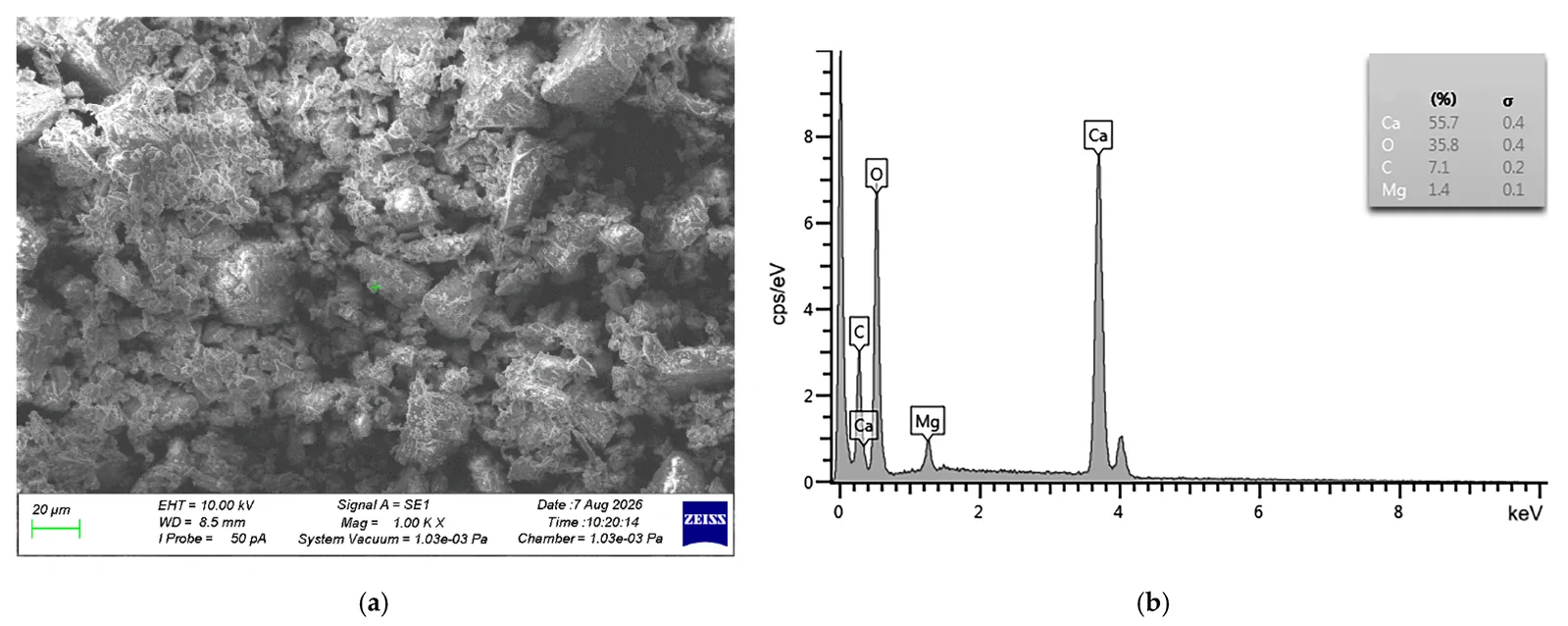
MC: (**a**) SEM image at 1000× magnification; (**b**) EDS spectrum.

6. Several types of fillers: GGBFS (Mechel, Chelyabinsk, Russia); quartz flour (QF) (NIKAS, Chelyabinsk, Russia); micro-calcite (MC) (NIKAS, Chelyabinsk, Russia). Table 1, Table 2 and Table 3 detail the chemical makeup and key characteristics of the fillers. Figure 2, Figure 3 and Figure 4 display the SEM and EDS analysis outcomes for GGBFS, QF, and MC particles.

The GGBFS particles exhibit an irregular, angular morphology, and their surfaces feature agglomerated smaller particles. Larger particles have an irregular geometric shape with smoothed edges. Small particles are also characterized by an irregular geometric shape, but with sharper edges.

Agglomerated clusters of smaller particles adhere to the surface of larger QF and MC particles, giving them an irregular angular shape. The surface of the particles is uneven and rough.

**Figure 5:**
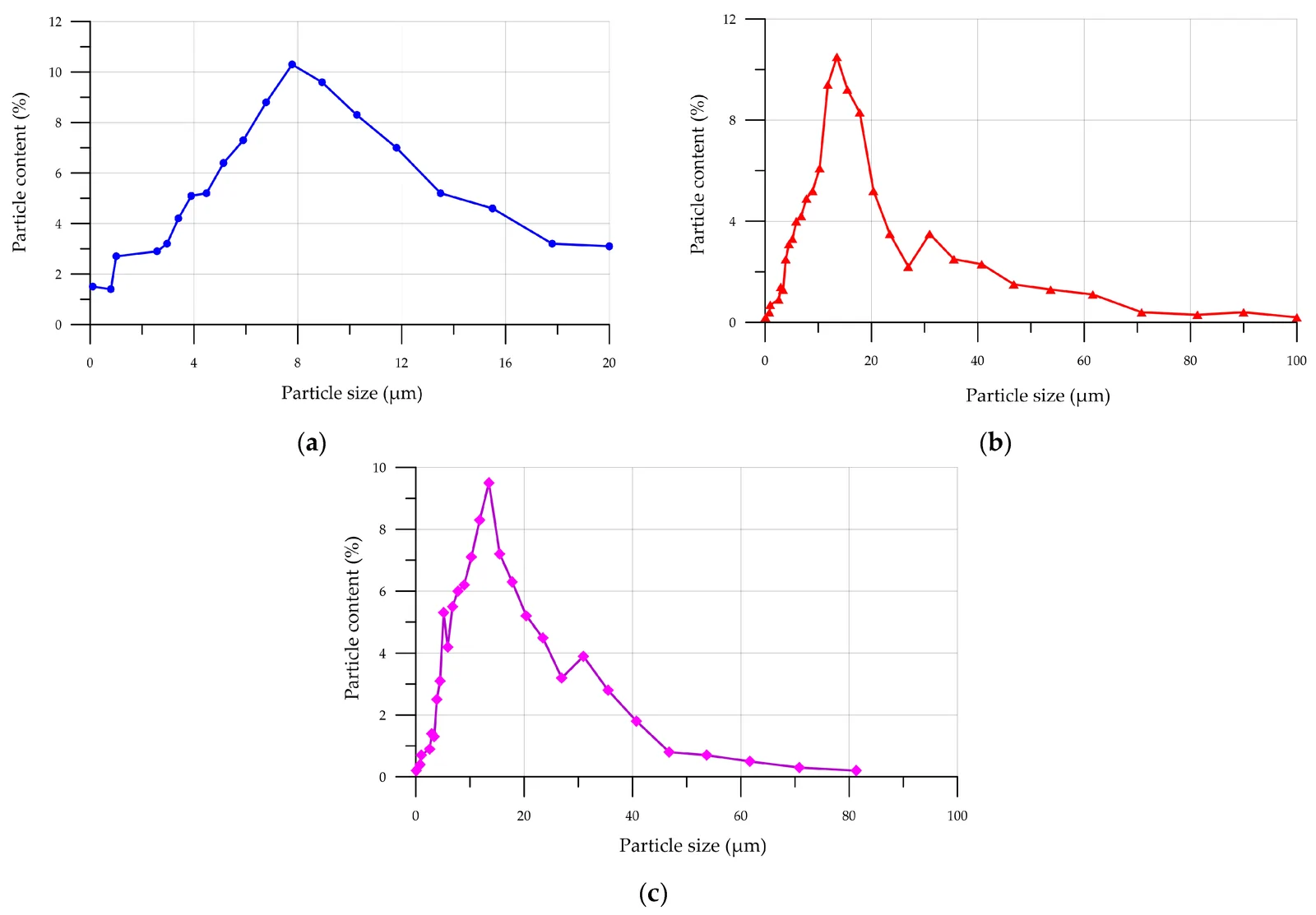
Particle size distribution curves: (**a**) QF; (**b**) MC; (**c**) GGBFS.

Figure 5 displays the particle size distribution curves for GGBFS, QF, and MC.

The following particle distribution is typical for mineral fillers:

– QF: 68.6% of the particles are in the 0.1 µm to 10 µm size range; the remaining 31.4% of the particles are in the 10 µm to 20 µm size range.
– MC: 32.1% of the particles are in the 0.1 µm to 10 µm size range; 60.4% of the particles are in the 10 µm to 21 µm size range; the remaining 7.5% of the particles are in the 21 µm to 100 µm size range.
– GGBFS: 37.7% of the GGBFS particles are in the 0.1 µm to 10 µm size range; 43.6% of the particles are in the 10 µm to 21 µm size range; particles ranging in size from 21 µm to 82 µm accounted for 18.7%.

### 2.2. Methods

In Table 4’s recipe, experimental PC samples using epoxy resin and different mineral fillers were created by following these steps.

Step 1. Measuring and preparing the correct amounts of raw materials. The coarse and fine aggregates, along with fillers, were dried until their weight remained constant. This was done in a lab drying oven set at a temperature of 50 ± 10 °C.

Step 2. First, the plasticizer was added to the resin and mixed vigorously, followed by the hardener. The epoxy resin, hardener, and plasticizer were mixed at 1000–1200 rpm for 30 s. Fillers (GGBFS, QF, MC) were then gradually added to the composition and mixed at a speed of up to 500 rpm for 60 s until a homogeneous composition free of agglomerates was achieved. Fine aggregate was then added, and the mixture was mixed for 120 s. After adding the coarse aggregate, the mixture was blended to a homogeneous consistency. The ambient temperature during the mixing of the epoxy resin with the curing agent, plasticizing additive, and mineral components was 25 ± 5 °C.

Stage 3. Experimental PC samples were cast in three layers, compacting each layer. After casting, the molds containing the fresh polymer composite were placed on a laboratory vibrating platform and compacted at a frequency of 2900 ± 100 vibrations per minute to remove air bubbles, increase the density of the mixture, and break up potential particle clusters.

Stage 4. For the remaining 27 days, the PC samples, after 24 h of hardening and removal from molds, underwent aging in a lab environment maintained at 25 ± 5 °C and 50 ± 10% relative humidity.

The optimal proportions by weight for the raw material components—epoxy resin, coarse aggregate, fine aggregate, and mineral filler—were established through initial experimental findings. The optimal mineral filler content was 22% by weight and remained constant for all experimental compositions. In preliminary experiments, GGBFS was used to determine the optimal content of the mineral filler. Based on the results of these experimental studies, the polymer concrete matrix without a mineral filler demonstrated lower strength properties; therefore, for further research, it was decided to focus on several types of mineral fillers and increase their number to three, with the possibility of combining them. Three compositions were prepared with different types of mineral fillers (GGBFS, QF, and MC), three compositions with combinations of mineral fillers (GGBFS + QF, GGBFS + MC, QF + MC), and one composition with a combination of all three types of fillers. The mineral filler content by mass remained constant across all experimental formulations. Combinations of mineral fillers in equal proportions were determined to be the most rational and were experimentally optimized. All polymer concrete mixtures were prepared following the four stages described above. Controlling the resin-to-solids ratio involved ensuring dosing accuracy. The resin, filler, and hardener were dosed with an accuracy of ±1% by mass, while the aggregates—sand and crushed stone—were dosed with an accuracy of ±2% by mass. The sequence, duration, and speed of mixing the raw components were also controlled. Formulation 1PC served as the baseline composition for comparing the experimental results.

**Figure 6:**
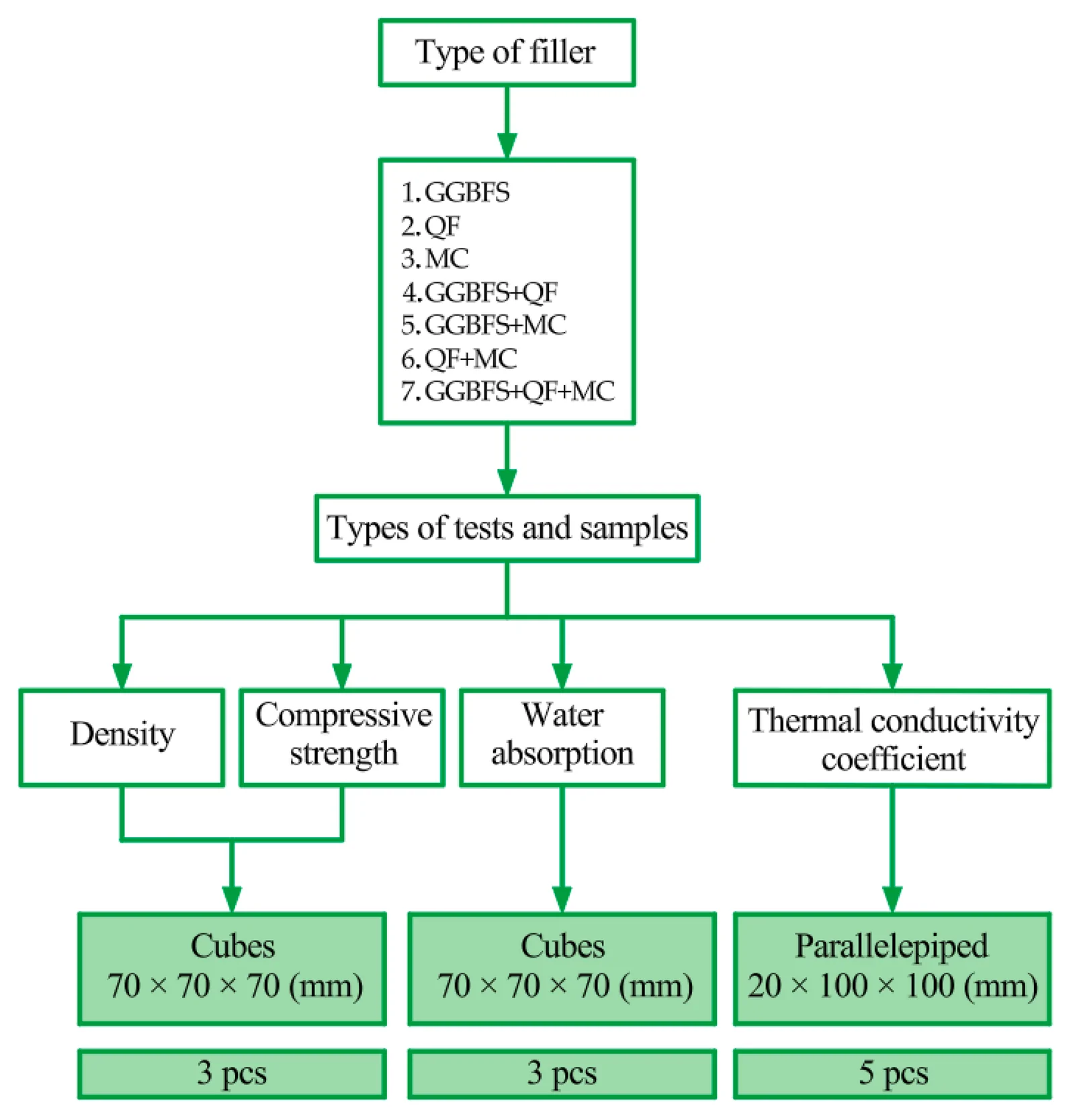
Experimental Study Program for PC Properties.

Figure 6 outlines the experimental study program designed to investigate the properties of PC with diverse mineral fillers.

Polymer concrete specimens were cured for 28 days under ambient laboratory conditions at a temperature of 25 ± 5 °C and a relative humidity of 50 ± 10%. For each mix, three 70 × 70 × 70 mm cubic specimens were tested to determine density and compressive strength, three 70 × 70 × 70 mm cubic specimens to determine water absorption, and five 100 × 100 × 20 mm parallelepiped specimens to determine thermal conductivity. A total of 42 cube specimens were prepared (21 for density and compressive strength testing, and 21 for water absorption testing) and 35 parallelepiped specimens for thermal conductivity testing. A total of 77 polymer concrete specimens were prepared.

The properties of PC were evaluated in accordance with the requirements of standardized methods.

1. Density *ρ_PC_* (kg/m^3^) of PC [47]. The density of PC was determined using 70 × 70 × 70 mm cube specimens aged for 28 days in an air-dry state at a temperature of 25 ± 5 °C and a relative humidity of 50 ± 10%. Density determination was carried out under similar conditions. The geometric dimensions of the specimens were first measured and then weighed. The density of each specimen was calculated with an error of up to 1 kg/m^3^ using Equation (1). PC density was determined by averaging the outcomes from three tests.
  (1) $$\rho_{\mathrm{PC}}=\frac{m}{V}\times 1000$$
  Here *m*—specimen mass (g); *V*—specimen volume (cm^3^).
2. Compressive strength *R_PC_* (MPa) [48,49,50,51,52]. The compressive strength of the PC was determined under laboratory conditions at a temperature of 25 ± 5 °C and a relative humidity of 50 ± 10%. To determine the compressive strength, 70 × 70 × 70 mm cube specimens were first mounted on the lower support plate of the testing equipment. The top plate of the apparatus was then aligned with the specimen’s support, and a force was applied at a speed of 0.6 ± 0.2 MPa/s until complete failure of the specimen, and the failure load was recorded. The compressive strength of each specimen was assessed using Equation (2) with the precision of 0.1 MPa. For each group of concrete samples, the strength was defined as the average of the top two strengths among three tested specimens:
  (2) $$R_{\mathrm{PC}}=\alpha \frac{F}{A}$$
  where *F* is the breaking load (N); *A* is the cross-sectional area of the specimen (mm^2^); *α* is a coefficient (for this case, *α* = 0.85).
3. Water absorption of *W_PC_* (%) [53]. The water absorption of PC was determined under laboratory conditions at a temperature of 25 ± 5 °C and a relative humidity of 50 ± 10%. Initially, the test involved placing 70 × 70 × 70 mm cube specimens in a container filled with water. The water was 20 degrees Celsius, with a margin of error of 2 degrees. After a 24-h soak in water, the PC specimens were weighed. The process of determining water absorption concluded when the last two weight measurements showed a difference of 0.1% or less. Formula (3) was used to determine the water absorption of the PC specimens, with a precision of 0.1%. To determine the water absorption of PC, the results from three specimens were arithmetically averaged:
  (3) $$W_{\mathrm{PC}}=\frac{m_{w}-m_{d}}{m_{d}}\times 100$$
  where *m_w_* is the mass of the water-saturated sample (g); *m_d_* is the mass of the dry sample (g).
4. Thermal conductivity coefficient λPC (W/m·K) [54]. The thermal conductivity coefficient of the PC was determined under laboratory conditions at a temperature of 25 ± 5 °C and a relative humidity of 50 ± 10%. An ITP-MG4 device from SKB Stroypribor, Chelyabinsk, Russia, was used to find the thermal conductivity coefficient PC. Initially, 100 × 100 × 20 mm parallelepiped samples underwent inspection to identify any parallelism discrepancies between their front surfaces. This deviation should not exceed ±0.5 mm. If deviations exceeded the norm, the samples were ground. Calipers were used to measure the sample thickness with an accuracy of ±0.1 mm. The measurements took place at four corners, each positioned (50 ± 5) mm from the vertex and the center of its respective edge. The samples underwent drying until reaching a constant mass after measurement. To find the thermal conductivity coefficient, the sample was dried and then inserted into the device. Five samples were tested, and their thermal conductivity was averaged to find the series’ mean.
5. Microstructure and EDS PC analysis were performed on a Zeiss EVO MA 18 electron microscope (Carl Zeiss Microscopy Ltd., Oberkochen, Germany) equipped with an integrated X-Max 50N energy-dispersive spectrometer (EDS) (Oxford Instruments, Abingdon, England). Images of the PC microstructure were obtained from the fracture surfaces of polymer concrete specimens. A section of the PC structure was examined at four different magnifications, ranging from lower to higher (50× to 3000×). Four SEM images were acquired for each composition studied. Pore dimensions were measured using software tools. Aztec software (version 4.0) was used to evaluate the elemental composition; an area of interest was selected on the specimen, and an X-ray spectrum was recorded over a period of 300 s.

## 3. Results and Discussion

### 3.1. Results of Evaluating the Properties of PC with Different Types of Mineral Fillers

**Figure 7:**
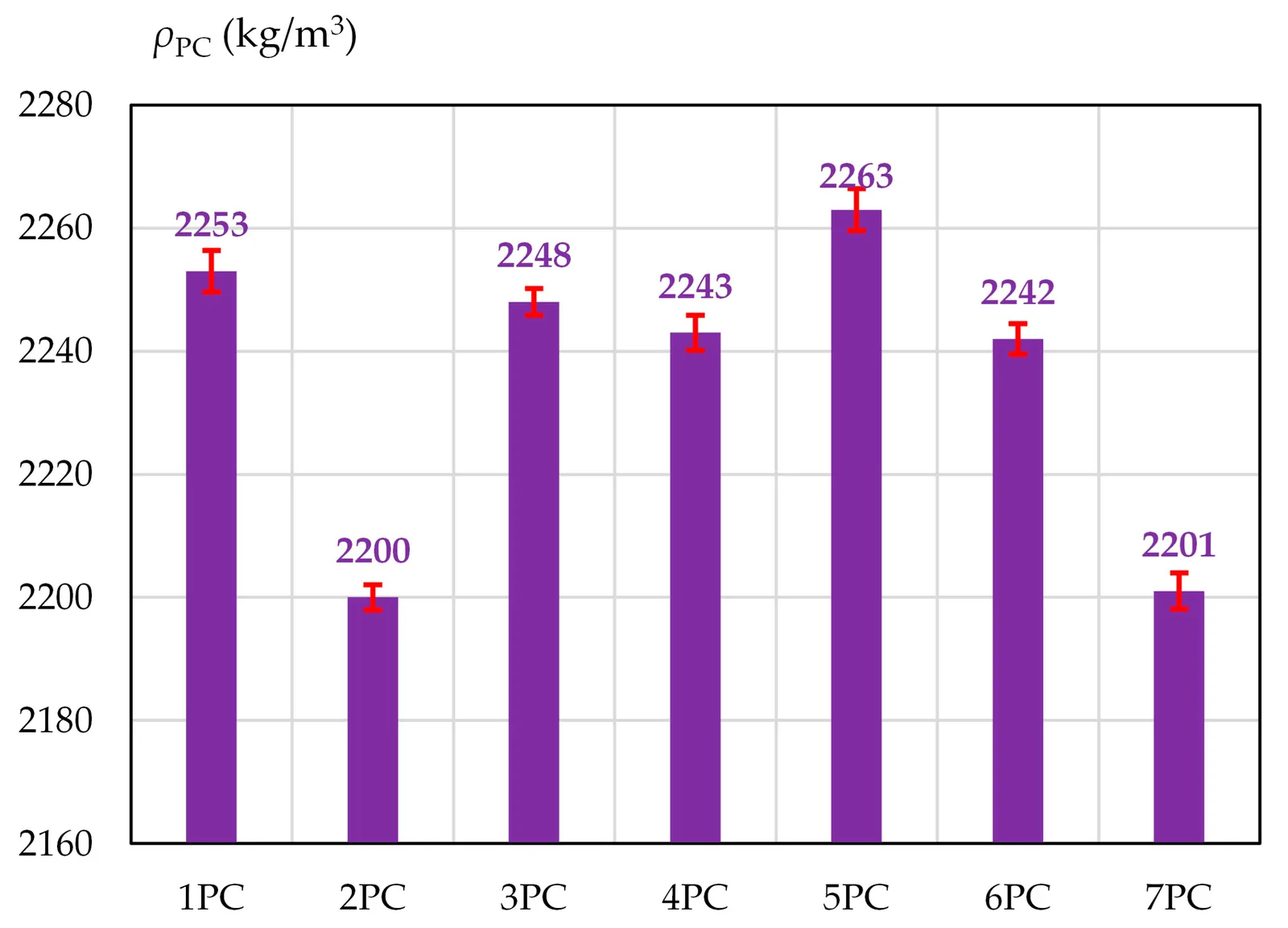
Density test results for PC samples (*ρ_PC_*).

The results of measuring the density (*ρ_PC_*) of experimental PC samples of compositions 1PC, 2PC, 3PC, 4PC, 5PC, 6PC, and 7PC are presented in Figure 7.

As can be seen from Figure 7, adding GGBFS to PC as a filler resulted in a density of 2253 kg/m^3^. For the analysis of experimental data, the composition of 1PC was adopted as the baseline for comparison. Throughout the text, percentage increases (+) and decreases (−) compared to composition 1PC are shown in parentheses after the actual value. Composition 2PC, which used QF as the mineral filler, had a lower density of 2200 kg/m^3^ (−2.4%) compared to composition 1PC, which is logical, as GGBFS particles are characterized by higher density. The 3PC composition with MC mineral filler had a density of 2248 kg/m^3^ (−0.2%), which is slightly lower than the baseline value. These density changes depending on the filler type are directly related to the density of the filler itself. Among the three types of mineral fillers used, GGBFS has the highest density of 2.81 g/cm^3^, QF has a density of 2.65 g/cm^3^, and MC has a density of 2.7 g/cm^3^. The density of 4PC composition with a combination of two types of mineral fillers, GGBFS + QF, was 2243 kg/m^3^ (−0.4%). Composition 5PC, which used a combination of GGBFS + MC fillers, had the highest density of 2263 kg/m^3^ (+0.4%). The samples of composition 6PC with a combination of QF + MC fillers had a density of 2242 kg/m^3^ (−0.5%). Polymer concrete of composition 7PC with a combination of three types of mineral fillers had a density of 2201 kg/m^3^ (−2.3%). The difference between the highest and lowest density values was 63 kg/m^3^.

**Figure 8:**
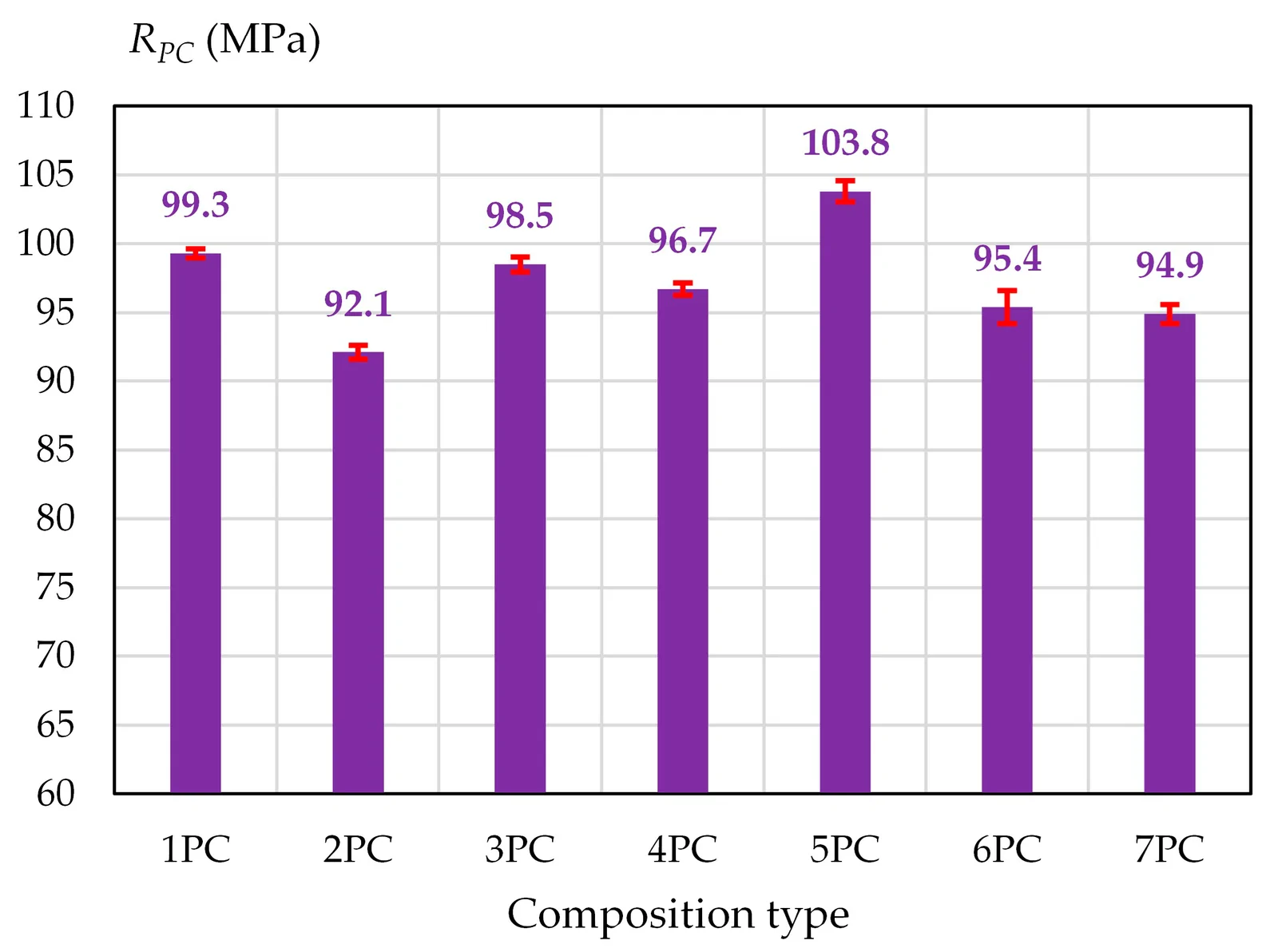
Compressive Strength Test Results (RPC) for PC Specimens.

Additionally, Figure 8 presents the compressive strength test results for PC (*R*_PC_) when different mineral fillers were used.

**Figure 9:**
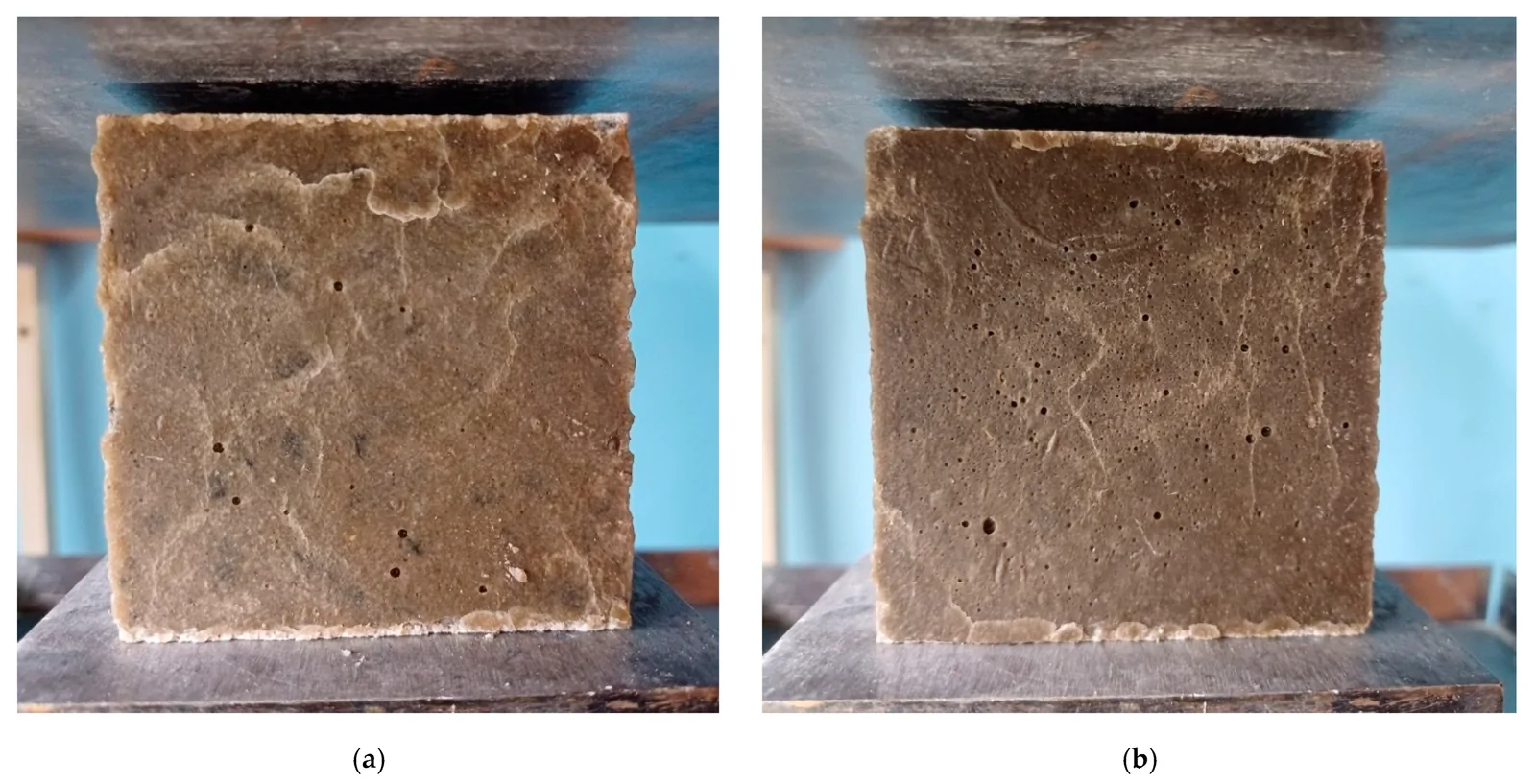
Polymer concrete specimens after determining compressive strength: (**a**) 2PC; (**b**) 5PC.

Composition 1PC, which used GGBFS as the mineral filler, had a compressive strength of 99.3 MPa, which is one of the best results. Composition 2PC with QF filler demonstrated the lowest compressive strength of 92.1 MPa (−7.3%) among all PC compositions. The compressive strength of composition 3PC with MC filler was 98.5 MPa (−0.8%), which is slightly lower than that of composition 1PC. Among all three types of PC, which used a single mineral filler, PC with GGBFS and MC demonstrated the highest performance. Composition 4PC with a combined GGBFS + QF filler has a compressive strength of 96.7 MPa (−2.6%). The greatest compressive strength of 103.8 MPa (+4.5%) was demonstrated by the composition with the GGBFS + MC combination. The QF + MC combination provided a compressive strength of 95.4 MPa (−3.9%) for the 6PC composition. For the PC composition with a combination of two types of mineral fillers, the GGBFS + MC combination demonstrated the greatest effectiveness, followed by the composition with the GGBFS + QF combination and the composition with the QF + MC combination. The composition with a combination of three types of fillers, GGBFS + QF + MC, had a compressive strength of 94.9 MPa (−4.4%), which is lower than the compressive strength of the compositions with a combination of two types of mineral fillers. In polymer concrete, structural integrity is a key factor and directly affects the density of the future composite. For example, if a polymer mixture is dominated by coarse and fine filler particles of identical size, large voids may form between them that the epoxy resin will not be able to fill, and weak zones may appear in the polymer composite, which subsequently reduce its strength [55]. The introduction of different types of mineral fillers (GGBFS, QF, MC) and their combinations, which have a polydisperse particle size distribution (Figure 5), into the polymer concrete mixture helps solve this problem. Smaller filler particles are located in the spaces between the larger ones and lead to the formation of a denser composite structure with fewer pores and, as a result, high compressive strength [56]. Fine filler particles interact with the epoxy resin and disperse within it, while the resulting mixture of resin and filler particles coats the fine and coarse aggregate grains. It is also important to note that GGBFS, QF, and MC within the epoxy matrix function primarily as physical fillers but can also participate in chemical reactions. At the molecular level, the interaction between the epoxy resin and the mineral filler particles involves a range of physical and chemical processes. Initially, van der Waals forces facilitate the adhesion of resin molecules to the mineral filler particles. Subsequently, the resin molecules adsorb onto the surface of the mineral particles, forming an interphase layer. Additionally, epoxy groups undergo chemical interactions with silanol groups (-Si-OH) on the GGBFS surface, forming strong bonds. The examined mineral filler types and their combinations demonstrate good performance, allowing the manufacture of composite concrete with a compressive strength exceeding 92.1 MPa. However, the GGBFS + MC combination is the best, achieving a maximum compressive strength of 103.8 MPa. Figure 9 below shows composite concrete samples after determining their compressive strength and analyzing their failure patterns.

The weaker 2PC composition is characterized by a greater number of cracks than the stronger 5PC composition. The main distinguishing features of the failure pattern of polymer concrete from traditional cement concrete are as follows. Polymer concrete is capable of accumulating deformations and redistributing stresses within the composite structure; therefore, it is characterized by greater plastic deformations before failure compared to traditional cement concrete [30,57,58,59]. The polymer concretes developed in this study are also characterized by the appearance of a crack network with partial retention of load-bearing capacity without localized failure, accompanied by delamination of parts of the composite.

**Figure 10:**
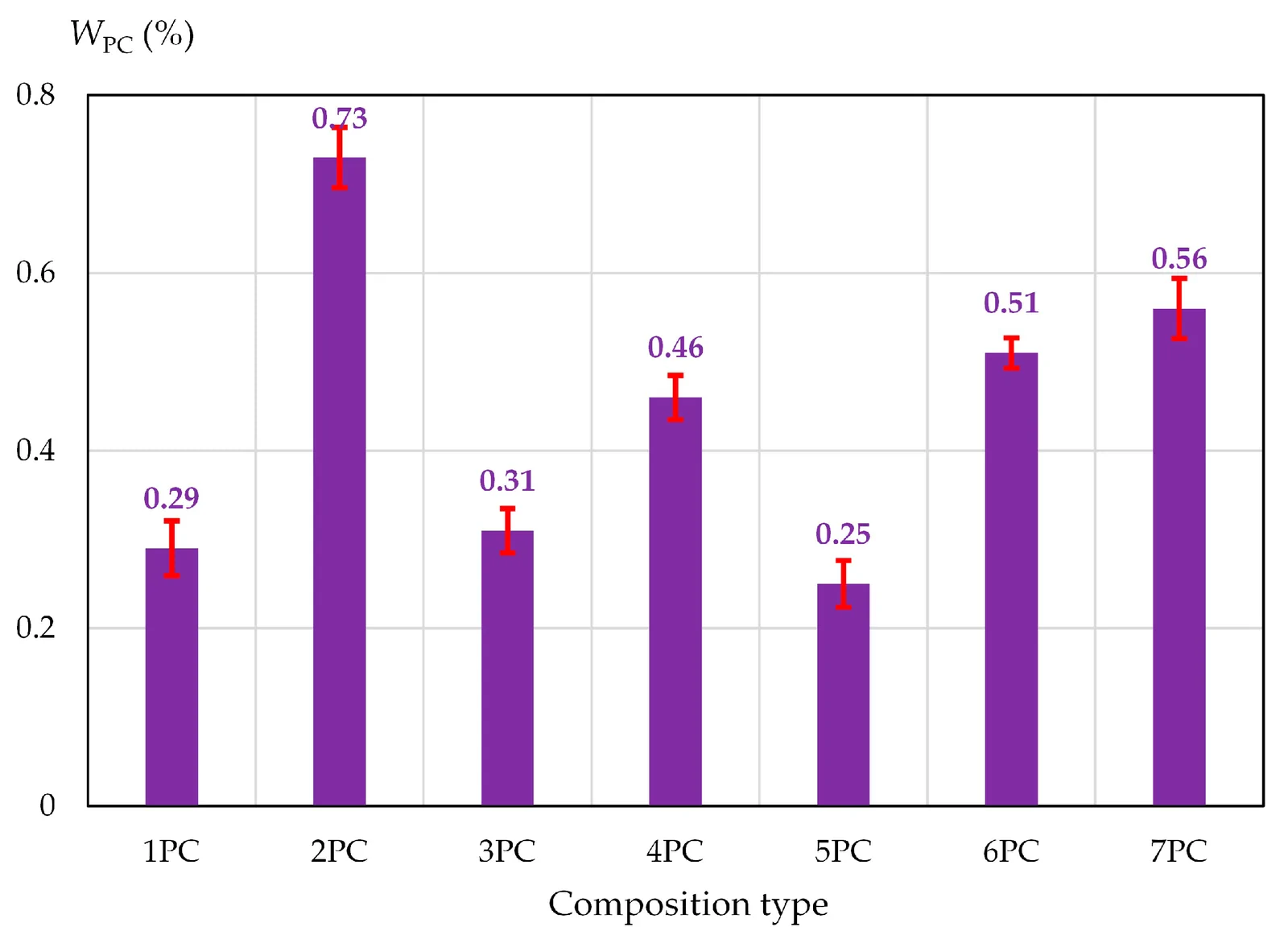
Water absorption test results for *W*_PC_ samples.

Figure 10 presents the results of measuring the water absorption (*W*_PC_) of the experimental polymer concrete specimens.

The water absorption of the 1PC composite with GGBFS filler was 0.29%. The 2PC composite, which used QF as the mineral filler, had a maximum water absorption of 0.73% (+151.7%). The 3PC composite with MC filler had a water absorption of 0.31% (+6.9%). Among composites with a single mineral filler, the 1PC and 3PC composites demonstrated the best results. The water absorption of the 4PC composite with a combination of GGBFS and QF fillers was 0.46% (+58.6%), which is slightly higher than that of the 1PC and 3PC composites. The lowest water absorption of 0.25% (−13.8%) was recorded for the 5PC composite with a combination of GGBFS and MC mineral fillers. For the 6PC and 7PC compositions with a combination of QF + MC and GGBFS + QF + MC, the water absorption values were 0.51% (+75.9%) and 0.56% (+93.1%). The type of filler in a polymer composite significantly affects its pore structure and, consequently, water absorption. PC with a more homogeneous polymer matrix and dense packing of mineral filler particles has a smaller number of pores and voids that can be filled with water. As a result, their water absorption will be lower in comparison with a composite in which the mineral filler particles are non-uniformly located and create greater voids in the polymer matrix. The granulometric characteristics of the fillers are also important. A polydisperse granulometric composition of the filler ensures better packing than fillers with a monofractional granulometric composition [55,60]. GGBFS and MC have a distinctly polydisperse particle size distribution, and their combination produces polymer concrete with the highest compressive strength of 103.8 MPa and the lowest water absorption of 0.25%. The shape and surface of the filler particles are important. Particles with an irregular angular shape and a rough surface are better wedged into the spaces between larger grains of fine and coarse aggregate.

**Figure 11:**
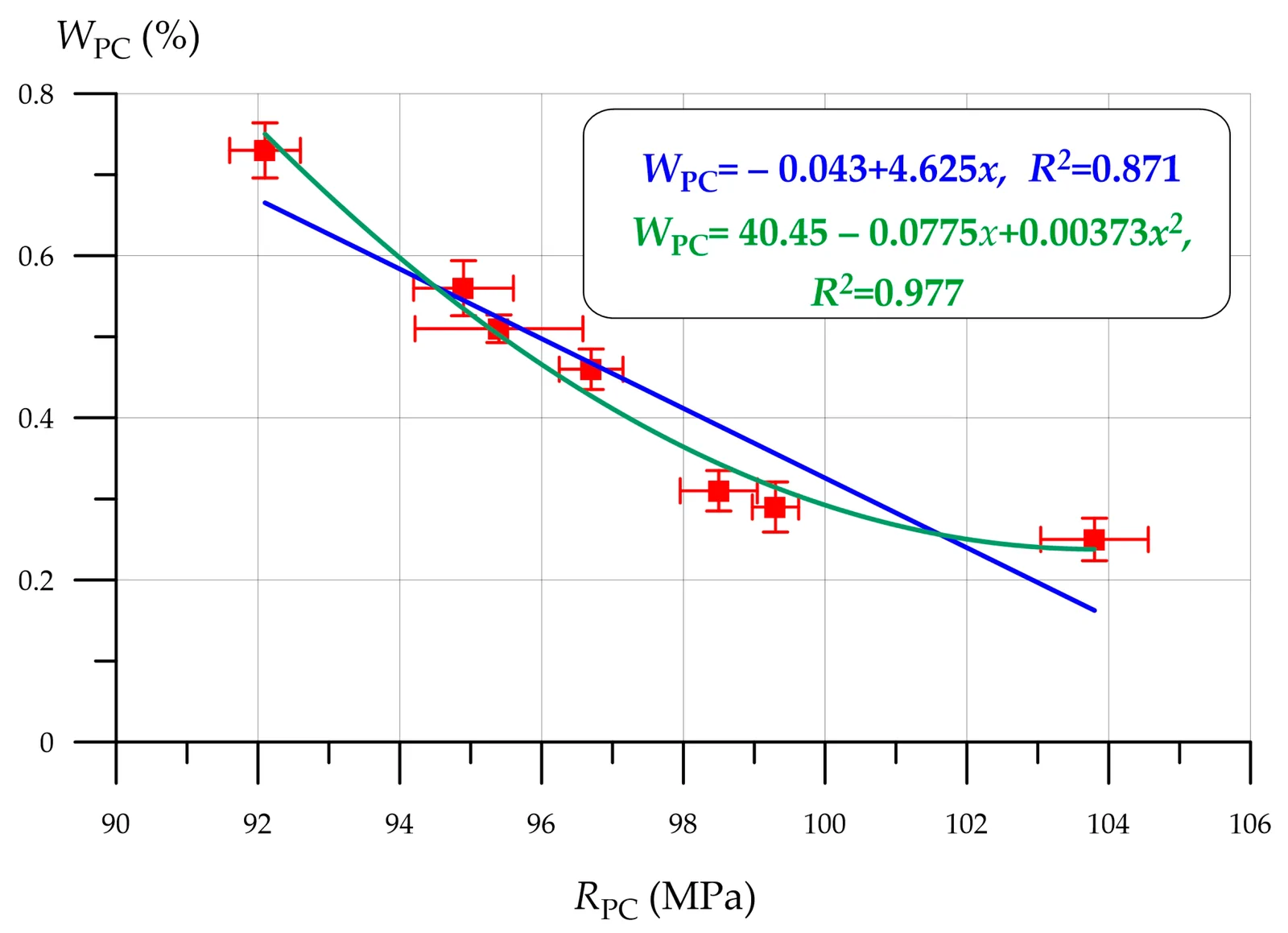
Regression between water absorption (*W*_PC_) and compressive strength (*R*_PC_) of polymer concrete.

The linear regression between water absorption and compressive strength of PC is shown in Figure 11.

Figure 11 demonstrates the approximation of the experimental data by a linear relationship and a second-degree polynomial:

*W_PC_* = −0.043 *x* + 4.625,   *R*^2^ = 0.871 (4)

*W_PC_*= 40.45 − 0.775 *x* + 0.00373 *x*^2^,   *R*^2^ = 0.977 (5)

The linear correlation between water absorption and compressive strength PC is strong, with a determination coefficient of *R*^2^ = 0.8711. The *p*-value = 0.002. Therefore, the relationship is significant (*p* < 0.05). Also shown is a second-degree polynomial fit, which significantly better describes the relationship between water absorption and clarity. As can be seen, *R*^2^ = 0.977, and the curve behaves well both in the middle of the interval and at the boundaries of the region under consideration. The established relationship is advisory in nature. The linear and polynomial relationships obtained (Formulas (3) and (4)) are applicable only to the present study and cannot be extrapolated to other studies.

**Figure 12:**
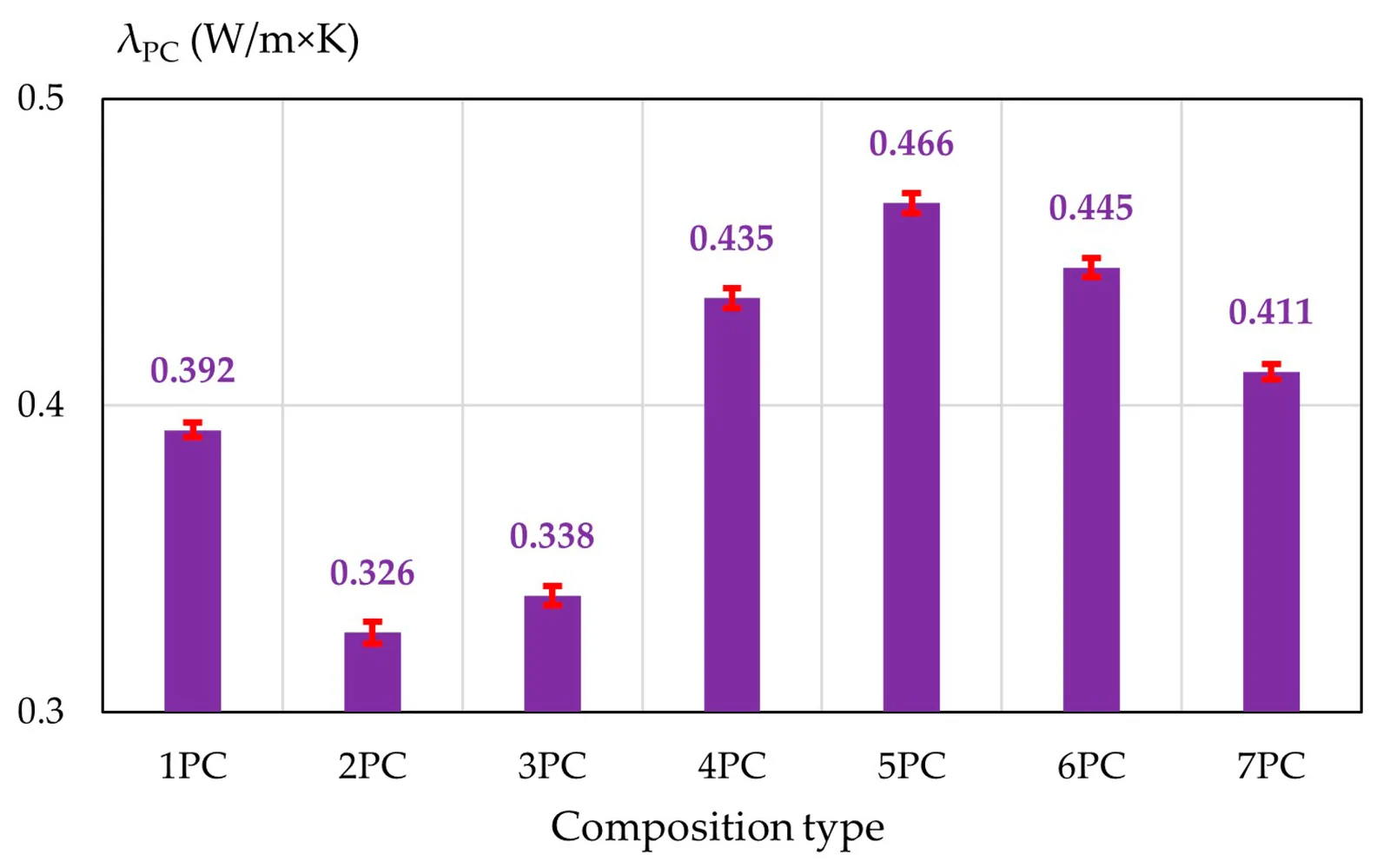
Thermal conductivity coefficient (λ_PC_) results for PC samples.

The results of measuring the thermal conductivity coefficients of polymer concrete (λ_PC_) with various types of mineral fillers are presented in Figure 12.

The thermal conductivity coefficient of 1PC composition with GGBFS filler was 0.392 W/m·K. 2PC and 3PC compositions with QF and MC mineral fillers had slightly lower thermal conductivity coefficients of 0.326 W/m·K (−16.8%) and 0.338 W/m·K (−13.8%), respectively. PC compositions with a combination of two or more types of mineral fillers are characterized by higher thermal conductivity coefficients. Thus, the 4PC composition with a combination of mineral fillers GGBFS + QF had a thermal conductivity coefficient of 0.435 W/m·K (+11.0%), the 5PC composition with GGBFS + MC had a thermal conductivity coefficient of 0.466 W/m·K (+18.9%), and for the 6PC composition its value was 0.445 W/m·K (+13.5%). The lowest thermal conductivity coefficient for compositions with combined mineral fillers was recorded for the 7PC composition with a combination of three types of mineral fillers GGBFS + QF + MC—0.411 W/m·K (+4.8%). The inclusion of mineral particles in the epoxy-polymer matrix contributes to an increase in its thermal conductivity. The epoxy-polymer matrix itself is a poor thermal conductor; however, the incorporation of mineral aggregates and fillers facilitates the creation of zones with directional heat flows. The mineral components of polymer concrete are capable of better heat transfer. The overall effect of incorporating various types of mineral fillers and their combinations is primarily determined by factors such as the particle size distribution of the filler particles and their arrangement within the polymer matrix. As can be seen, polymer concrete compositions with a single type of mineral filler have lower thermal conductivity coefficients, while compositions combining two or more types of mineral fillers typically have higher thermal conductivity coefficients. This fact indicates that combining several types of mineral fillers creates a greater number of zones through which heat will spread more effectively within the polymer concrete matrix [61,62]. However, it is important to consider the presence of pores in the polymer matrix structure, which reduce heat transfer and can offset this effect. The mineral fillers GGBFS, QF, and MC possess different thermal conductivities, which influences the pattern of heat flow propagation within the polymer concrete structure. When combining two or more types of mineral fillers with differing particle shapes and densities, achieving a uniform distribution throughout the composite volume during PC fabrication is technically more challenging; consequently, localized zones of higher density and thermal conductivity may form, thereby increasing the overall thermal conductivity of the composite. Furthermore, mixing multiple types of mineral particles creates various types of contact zones within the continuous filler particle network, facilitating heat transfer through the polymer matrix. The higher thermal conductivity coefficient observed in the 5PC composition is attributable to the aforementioned effects. The GGBFS + MC particles form a continuous network of mineral particles that enhances thermal conductivity, while their polydisperse particle size distribution promotes the formation of a denser structure with reduced porosity [63].

**Figure 13:**
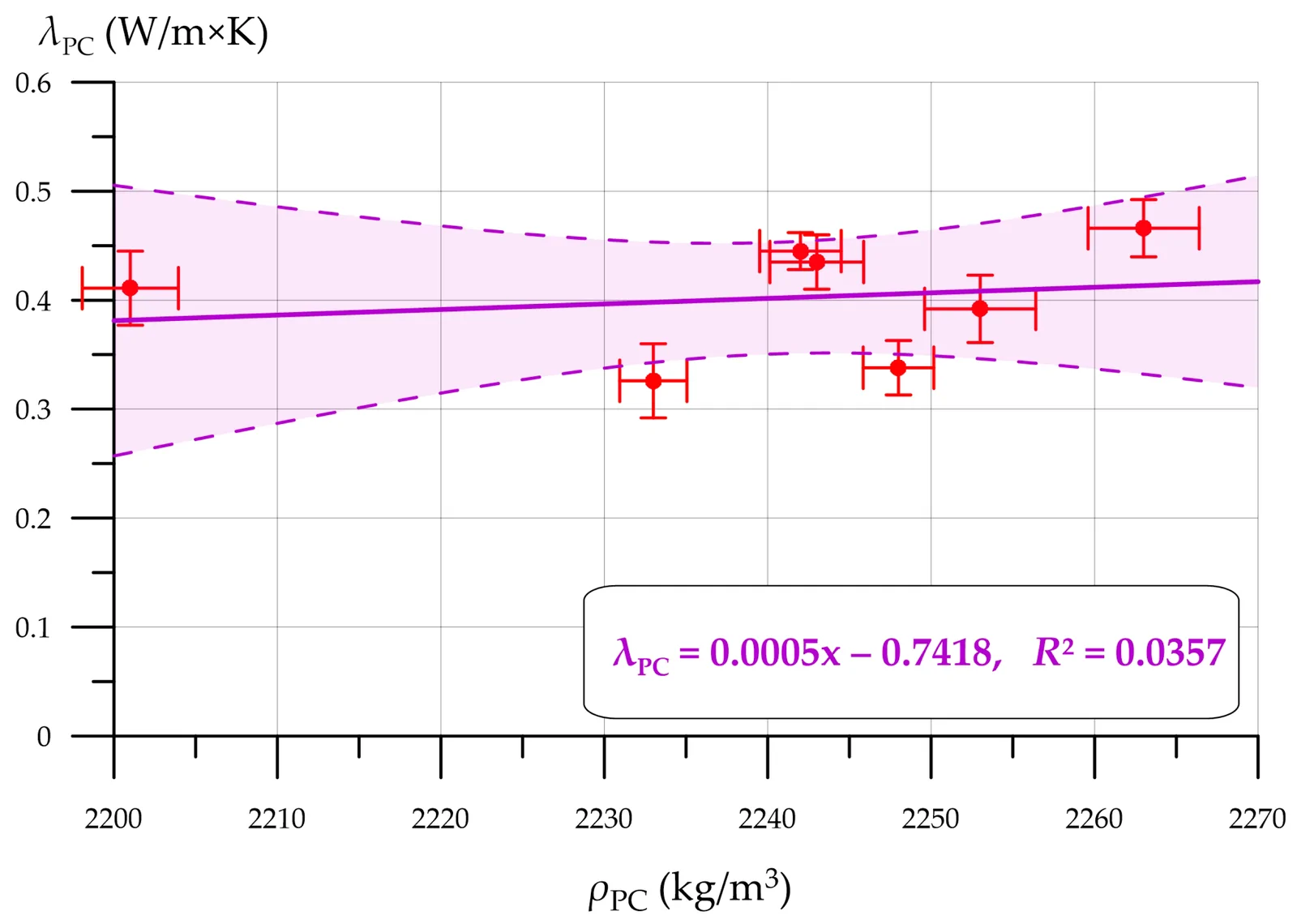
Linear regression between thermal conductivity (*λ_PC_*) and density (*ρ_PC_*) of PC.

Figure 13 shows the linear regression between the thermal conductivity coefficient (*λ_PC_*) and the density (*ρ_PC_*) of polymer concrete.

The correlation between thermal conductivity *λ_PC_* and density *ρ_PC_* is weak, with a determination coefficient of (*R*^2^ = 0.0357). The *p* value is *p* > 0.05, indicating that the relationship between these properties is not significant.

A summary table of the standard deviations (*σ*) and coefficients of variation (*V*) for each of the determined properties of the RS is presented below (Table 5).

The primary criteria for determining the optimal polymer concrete composition were the actual property values of the composites: density, compressive strength, water absorption, and thermal conductivity. Composition 5PC exhibited the highest compressive strength and the lowest water absorption. Although 5PC also had the highest thermal conductivity, it was identified as the optimal composition because it outperformed the others (1PC, 2PC, 3PC, 4PC, 6PC, 7PC) based on two of the three criteria.

Based on the experimental results obtained, the effects of modifying the RS with various mineral fillers and the contribution of each combination are as follows:

– Composition 1PC (22% GGBFS). This is the most effective among the three compositions (1PC, 2PC, 3PC) utilizing a single type of mineral filler. It exhibited the highest compressive strength (99.3 MPa) and the lowest water absorption (0.29%), meeting two of the three criteria established to evaluate the effectiveness of the mix design. This composition also displayed the highest thermal conductivity coefficient among the three single-filler compositions.
– Composition 2PC (22% QF). This composition is the least effective of the three single-filler compositions. The compressive strength was 92.1 MPa, and water absorption was 0.73%. However, it recorded the lowest thermal conductivity coefficient among the three single-filler compositions (0.326 W/m·K).
– Composition 3PC (22% MC). This composition ranks second in effectiveness among the three single-filler compositions. The compressive strength was 98.5 MPa, water absorption was 0.31%, and the thermal conductivity coefficient was 0.338 W/m·K.
– Composition 4PC (11% GGBFS + 11% QF). This ranks second in effectiveness among the three compositions utilizing a combination of two mineral fillers (4PC, 5PC, 6PC). However, it is less effective than compositions 1PC and 3PC. The compressive strength was 96.7 MPa, water absorption was 0.46%, and the thermal conductivity coefficient was 0.435 W/m·K.
– Composition 5PC (11% GGBFS + 11% MC). It exhibits the highest compressive strength (103.8 MPa) and the lowest water absorption (0.25%), with a thermal conductivity coefficient of 0.466 W/m·K. It was determined to be the most optimal among all seven experimental RS compositions.
– Composition 6PC (11% QF + 11% MC). This composition is the least effective among the three compositions combining two types of mineral fillers. The compressive strength was 95.4 MPa, water absorption was 0.51%, and the thermal conductivity coefficient was 0.445 W/m·K.
– Composition 7PC (7.4% GGBFS + 7.3% QF + 7.3% MC). Compared to compositions with a single type of mineral filler and those with a combination of two types—with the exception of composition 2PC—it exhibits lower compressive strength (94.9 MPa) and the highest water absorption (0.56%).
– Compositions with a single type of mineral filler are characterized by lower thermal conductivity coefficients compared to compositions with combinations of mineral fillers.

Table 6 below presents the results of the ANOVA performed on the data from determining the properties of PC of various compositions.

Analysis of variance (ANOVA) results show no significant differences in PC density values: *F* = 0.17; *p* = 0.842 (exceeding the 0.05 significance level). Density variations are directly related to the density of the mineral filler used. The highest density values were recorded for PC mixes containing GGBFS and MC fillers, which have higher densities than QF. A difference of 63 kg/m^3^ was observed between the highest and lowest density values among the 2PC and 5PC mixes. Compressive strength values differ in a statistically significant manner: *F* (2; 12) = 18.02; *p* < 0.001 (below the 0.05 significance level). Thus, the statistically significant differences identified via ANOVA confirm that the type of mineral filler has the greatest impact on this parameter. ANOVA results indicate no significant differences in PC water absorption values: *F* = 3.12; *p* = 0.081 (exceeding the 0.05 significance level). Similarly, no significant differences were found for PC thermal conductivity coefficients: *F* = 0.77; *p* = 0.553 (exceeding the 0.05 significance level).

In conclusion, the mineral fillers and combinations thereof examined in this study have the greatest influence on polymer concrete strength. The absence of statistically significant differences regarding properties such as density, water absorption, and thermal conductivity in this experiment indicates that the types of mineral fillers and combinations used do not affect these properties to the same extent as they affect compressive strength.

### 3.2. Results of SEM Analysis of the PC Structure and Discussion

**Figure 14:**
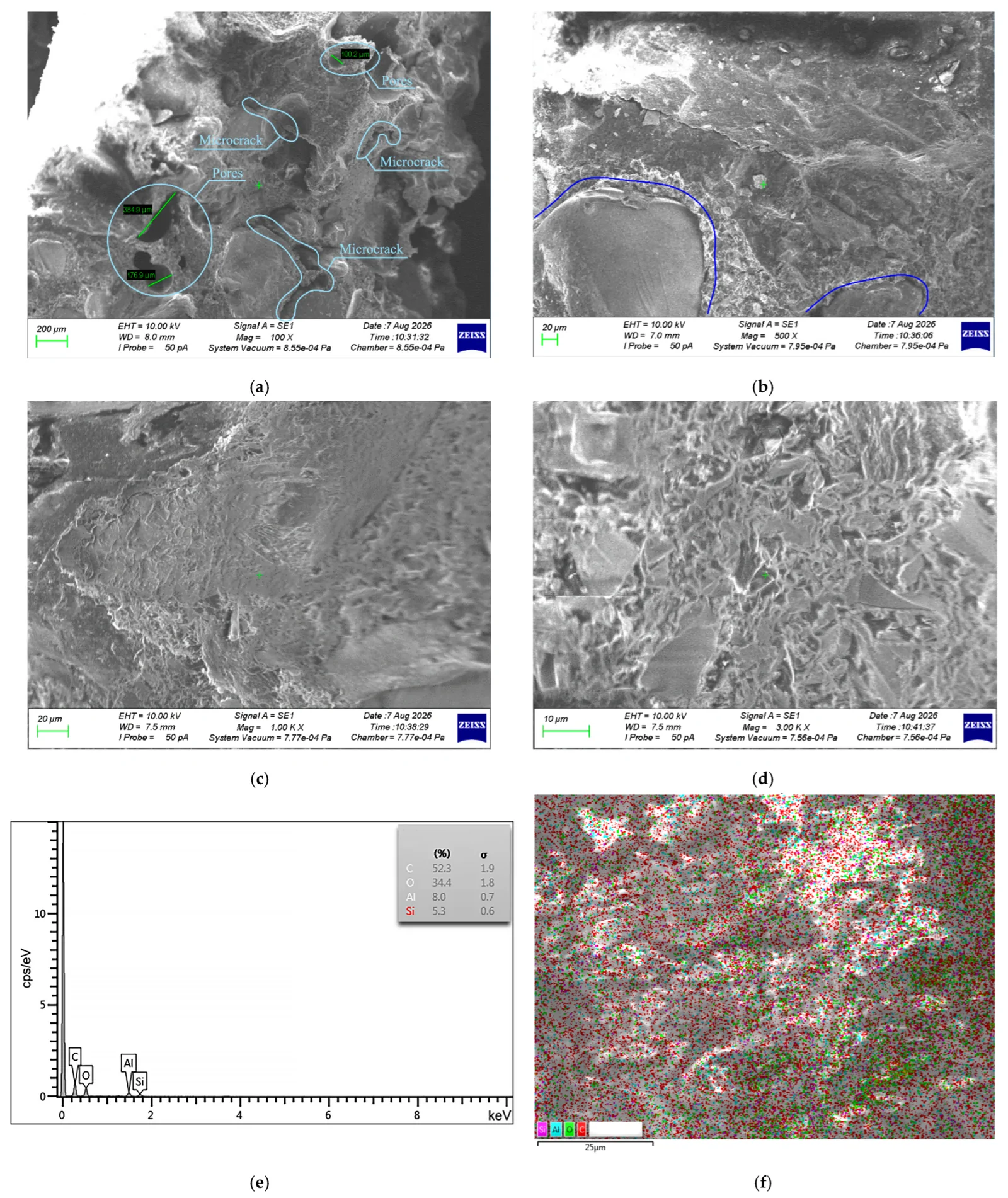
SEM images of the structure and EDS of 1PC composition with magnifications of: (**a**) 100×; (**b**) 500×; (**c**) 1000×; (**d**) 3000×; (**e**) spectrum; (**f**) multilayer EDS map.

**Figure 15:**
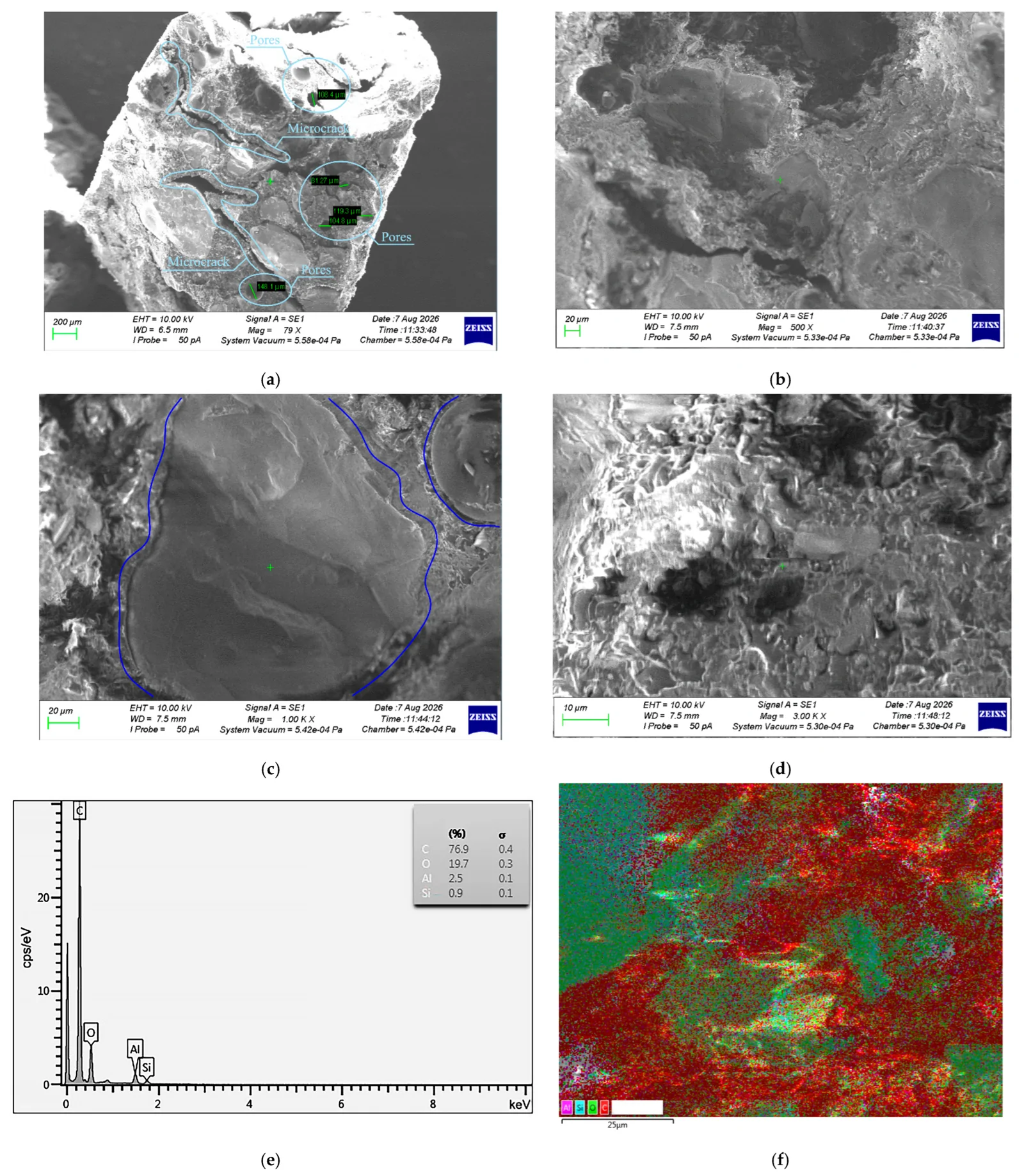
SEM images of the structure and EDS of 5PC composition with magnifications of: (**a**) 79×; (**b**) 500×; (**c**) 1000×; (**d**) 3000×; (**e**) spectrum; (**f**) multilayer EDS map.

**Figure 16:**
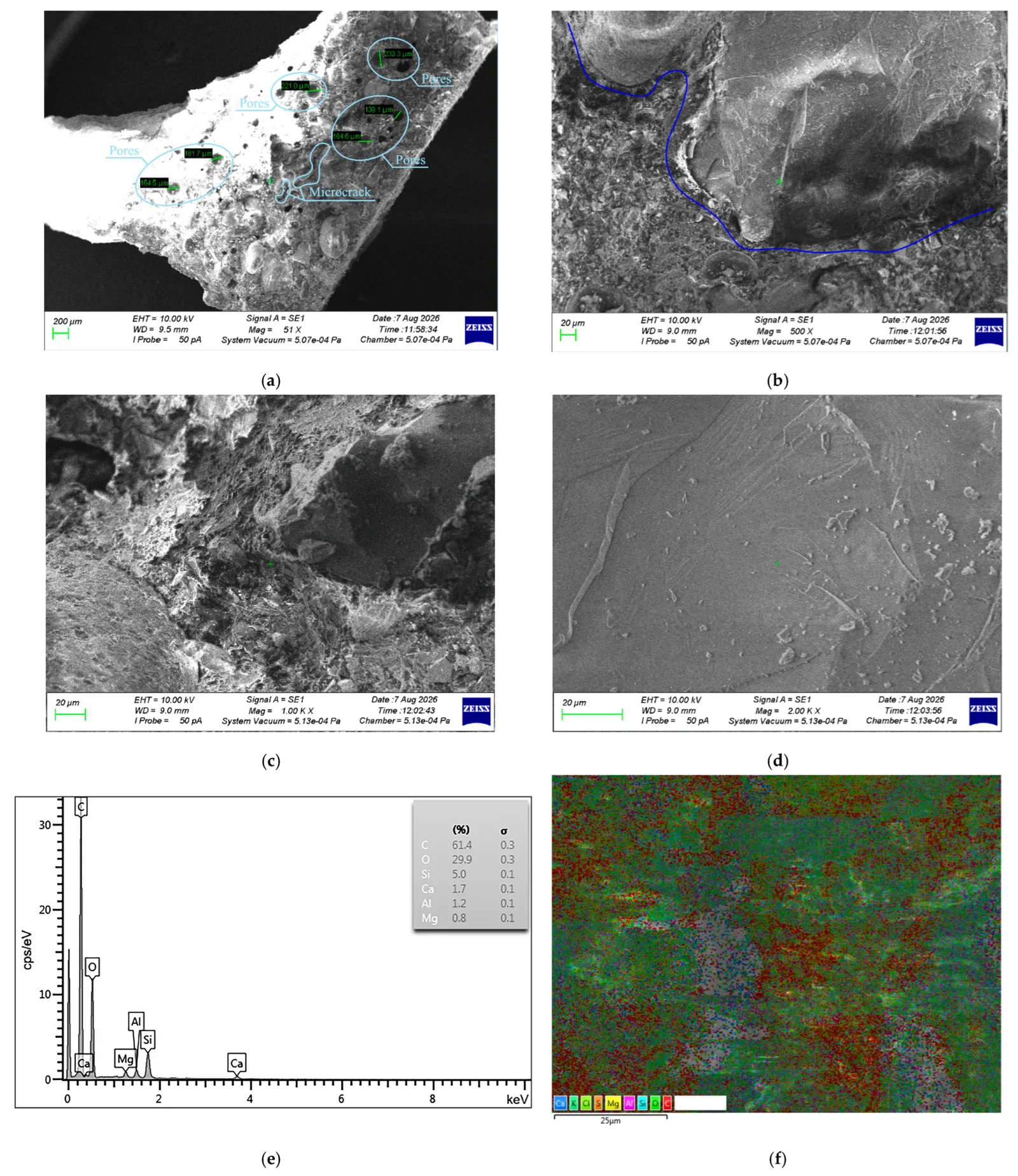
SEM images of the structure and EDS of 7PC at magnifications of: (**a**) 50×; (**b**) 500×; (**c**) 2000×; (**d**) 3000× (with markings); (**e**) spectrum; (**f**) multilayer EDS map.

A structural comparison was conducted on hardened PC samples of compositions 1PC, 5PC, and 7PC. Figure 14, Figure 15 and Figure 16 show morphological pictures of the PC structure and EDS maps reflecting the distribution of raw components in the epoxy-polymer matrix. Figure 14 shows SEM photos of the structure and EDS maps of composition 1PC at various magnifications.

Microstructural analysis of 1PC polymer concrete specimens focuses on the fracture surfaces. Images in Figure 14a–d illustrate the mechanism of crack initiation and propagation in the polymer matrix structure. Microcracks are primarily found within the phase interface zones. Clear interface boundaries between the filler grains and the polymer matrix are visualized. In Figure 14b, the phase interface appears as a smooth line with a rough surface. No microcracks are observed along the entire length of the interphase zone. Pores ranging in size from 100.2 µm to 384.9 µm are also identified in the polymer’s structure matrix of 1PC composition. The pores within the polymer matrix structure are predominantly spherical in shape. In the left corner of Figure 14a, two interconnected pores of different sizes—one smaller and one larger—are visible. EDS analysis revealed the presence of C, Al, O, and Si. The multilayer EDS map shows a fairly uniform distribution of the identified chemical elements.

SEM images illustrating the structure and EDS analysis of the 5PC composition at various magnifications are presented in Figure 15.

Figure 15a–d show the fracture surfaces of polymer concrete of composition 5PC, which characterize the development of cracks in its structure. Microcracks are also concentrated in zones at the phase boundaries and extend through the polymer matrix, bending around the filler grains. The phase interface in Figure 15c is also represented by a smooth line and has a rough surface. Delamination is observed along the right side of the transition zone surrounding the aggregate grain. The polymer matrix contains pores ranging in size from 81.27 µm to 148.1 µm. The pores are spherical in shape and randomly distributed within the polymer matrix structure. The following elements are identified based on the EDS results: C, Al, O, and Si. In the multilayer EDS map, elements O and C are located as distinct, uneven zones.

Different magnifications are used in Figure 16 to show the SEM images of the 7PC composition’s structure and EDS.

On the fracture surface (Figure 16a) of 7PC polymer concrete, spherical pores ranging in size from 139 µm to 233.3 µm are observed in the polymer matrix structure. Microcracks and distinct phase boundaries between the polymer matrix and filler grains are visible. The phase interface has a rough surface that adheres tightly to the aggregate grain along its entire length, with a single zone containing a small pore. The following elements are identified based on the EDS results: Ca, K, Cl, S, Mg, Al, Si, O, and C. The multilayer EDS map shows distinct zones of O, C, and Ca.

The microstructural analysis of PC with diverse compositions leads to these overall conclusions. All studied PC compositions are characterized by the presence of pores in the polymer matrix structure. The polymer composite structure itself is homogeneous, with mineral filler particles and aggregate grains in the polymer binder fairly evenly distributed. Microcracks are predominantly concentrated in the interface zones, indicating a similar failure pattern for polymer concretes of 1PC, 5PC, and 7PC compositions. Multilayer EDS maps demonstrate the distribution of particles of mineral components of various types in the structure of the polymer matrix. Overall, the distribution of Si, Al, and Ca elements is fairly uniform throughout the entire volume of the studied samples and indicates the homogeneity of the structure.

The effect of mineral filler type on the performance properties of polymer composites has been studied by other researchers. Table 7 presents an analysis of such studies.

The results of the comparative analysis with the results of other researchers (Table 6) show that the type, physical and mechanical properties, and granulometric composition of fillers and aggregates significantly affect the properties of polymer composites [72]. Optimum combinations of coarse and fine fillers and aggregates ensure dense packing of particles in the polymer matrix and directly affect the future properties of the composite [73,74]. The results obtained in this work also confirm that the type of mineral filler affects the structure and main performance properties of polymer concrete. Images of the microstructure of polymer concrete samples, presented in Figure 14, Figure 15 and Figure 16, clearly demonstrate the morphology at the phase boundary between the epoxy-polymer matrix and inclusions of mineral fillers. The polymer binder flows around the filler from all sides and creates a smooth boundary with a tight fit. The inclusion of smaller, irregularly shaped, angular particles of GGBFS, QF, and MC mineral fillers between coarse and fine aggregate grains promotes the formation of a larger number of transition zones. The presence of these so-called transition zones alters the trajectories of crack propagation in the composite structure when subjected to destructive loads. The overall crack path increases, bending around the aggregate and filler grains and absorbing greater energy. Thus, the optimization of the polymer concrete composition by introducing mineral fillers allows for the production of a polymer composite with a homogeneous structure and high strength properties. Among all the developed polymer concrete compositions, composition 5PC, with a combination of two types of GGBFS + MC fillers, demonstrates the best performance properties and is recommended as the optimal choice for future research. The combined modification of the epoxy matrix using two types of mineral fillers—GGBFS and MC—yields optimal performance due to the following effects. This combination of mineral particles with a polydisperse particle-size distribution ensures the formation of a homogeneous, dense polymer composite structure with reduced porosity. This study focused on the density, compressive strength, water absorption, thermal conductivity, and microstructural properties of polymer concretes with various types of mineral fillers and their combinations. A traditional optimization process was carried out by varying types of mineral fillers and selecting an optimal formulation to produce a polymer composite with enhanced strength properties. However, as this approach limits the scope of the study, future work on polymer concrete mix design will incorporate artificial intelligence tools to reduce the volume of experimental testing and enable a more detailed analysis of the interaction mechanisms between raw materials within the epoxy matrix [75,76]. Future research plans include durability testing according to the following program: assessing the chemical resistance coefficient; determining frost resistance; and determining water resistance. In addition, the use of in situ AFM-based nanomechanical indentation methods is planned to observe nano- and microcracks at the filler–resin interface during load application [77].

The polymer concretes obtained in this study have potential applications in mechanical engineering as well as in industrial and civil construction, provided their properties meet design requirements. Full-scale practical application of the developed polymer concretes in these sectors will be possible following extensive experimental durability testing. Should industrial-scale production be undertaken, it is essential to adopt a comprehensive approach and optimize all manufacturing processes to minimize environmental impact.

## 4. Conclusions

This study determined the compressive strength, density, water absorption, and thermal conductivity of PC produced from epoxy resin, quartz sand, crushed granite, and several types of mineral fillers (GGBFS, QF, and MC), with an emphasis on the composite structure.

(1) The density of polymer concrete varies depending on the type of mineral filler and their combinations. The highest density was recorded for the 5PC composition with a combination of two types of mineral fillers (GGBFS + MC). The lowest density was recorded for the 2PC composition with QF.
(2) The GGBFS + MC filler combination is the most effective, providing the highest compressive strength of 103.8 MPa among all polymer concrete compositions. The 2PC composition with QF has the minimal strength at 92.1 MPa.
(3) Polymer concrete composition 5PC with a combination of mineral fillers GGBFS + MC has the smallest water absorption of 0.25%. The highest water absorption value of 0.73% is characteristic of composition 2PC with QF.
(4) The lowest value of thermal conductivity coefficient of 0.326 W/m·K is characteristic of composition 2PC. PC compositions with a combination of two or more types of mineral fillers are characterized by higher values of thermal conductivity coefficients. Composition 5PC has the highest value of thermal conductivity coefficient of 0.466 W/m·K.
(5) The types of mineral fillers and their combinations considered in this study demonstrate good efficiency and make it possible to obtain PC with compressive strength from 92.1 MPa to 103.8 MPa and low water absorption. The formulation of composition 5PC is the most optimal: ER—15%; HP—1.5%; P—1.5%; QS—30%; CrS—30%; GGBFS—11%; MC—11%.
(6) Polymer concretes have similar morphologies. The structure of polymer concrete is uniform, with a randomly distributed pore size ranging from 81.27 µm to 384.9 µm. Microcracks are predominantly concentrated in the phase boundaries and extend through the polymer matrix, enveloping the filler grains. The polymer matrix–filler grain interfaces are clearly visible. Multilayer EDS maps of polymer concrete visually demonstrate the distribution of mineral components in the polymer matrix.
(7) Modifying polymer concrete with mineral fillers and combinations thereof facilitates the formation of a homogeneous material structure with reduced porosity.
(8) The developed polymer concretes are characterized by high compressive strength and low water absorption; they show potential for practical application in mechanical engineering as well as in industrial and civil construction, subject to durability testing and the determination of the full range of required properties.

## Figures and Tables

**Table 1:** GGBFS Properties.

CaO (%)	SiO_2_ (%)	Al_2_O_3_ (%)	MgO (%)	FeO (%)	Na_2_O (%)	K_2_O (%)	TiO_2_ (%)	MnO (%)	S (%)	Chloride Ion (%)
40.3	37.8	6.8	12.1	0.2	1.1	0.8	0.1	0.5	0.2	0.1
Density (g/cm^3^)					2.81					
Moisture (wt. %)					0.1					
Specific surface area (m^2^/kg)					0.48					

**Table 2:** QF properties.

SiO_2_ (%)	CaO (%)	LOI (%)
99.5	0.1	0.4
Density (g/cm^3^)		2.65
Moisture (wt. %)		0.2
Specific surface area (m^2^/kg)		1.2

**Table 3:** MC properties.

CaCO_3_ (%)	MgCO_3_ (%)	Fe_2_O_3_ (%)	SiO_2_ (%)	Al_2_O_3_ (%)
98.24	0.9	0.05	0.79	0.02
Density (g/cm^3^)		2.7		
Moisture (wt. %)		0.2		
Specific surface area (m^2^/kg)		0.49		

**Table 4:** Ratio of PC components (wt. %).

Composition Type	Type of Mineral Filler	ER (%)	HP (%)	P (%)	QS (%)	CrS (%)	GGBFS (%)	QF (%)	MC (%)
1PC	GGBFS	15	1.5	1.5	30	30	22	0	0
2PC	QF	15	1.5	1.5	30	30	0	22	0
3PC	MC	15	1.5	1.5	30	30	0	0	22
4PC	GGBFS + QF	15	1.5	1.5	30	30	11	11	0
5PC	GGBFS + MC	15	1.5	1.5	30	30	11	0	11
6PC	QF + MC	15	1.5	1.5	30	30	0	11	11
7PC	GGBFS + QF + MC	15	1.5	1.5	30	30	7.4	7.3	7.3

1PC—polymer concrete with a single type of mineral filler GGBFS. 2PC—polymer concrete with a single type of mineral filler QF. 3PC—polymer concrete with a single type of mineral filler MC. 4PC—polymer concrete with two types of mineral fillers GGBFS and QF. 5PC—polymer concrete with two types of mineral fillers GGBFS и MC. 6PC—polymer concrete with two types of mineral fillers QF и MC. 7PC—polymer concrete with three types of mineral fillers GGBFS, QF и MC.

**Table 5:** Statistical characteristics.

Composition Type	Density		Compressive Strength		Water Absorption		Thermal Conductivity Coefficient	
	*σ*	*V*	*σ*	*V*	*σ*	*V*	*σ*	*V*
1PC	3.39	0.15	0.32	0.33	0.03	10.8	0.0024	0.62
2PC	2.05	0.09	0.49	0.54	0.03	4.68	0.0035	1.08
3PC	2.16	0.09	0.54	0.55	0.02	7.96	0.0032	0.94
4PC	2.87	0.13	0.45	0.46	0.02	5.46	0.0034	0.78
5PC	3.39	0.15	0.76	0.73	0.03	10.6	0.0032	0.68
6PC	2.49	0.11	1.18	1.25	0.02	3.35	0.0033	0.73
7PC	2.94	0.13	0.7	0.74	0.03	6.1	0.0024	0.59

**Table 6:** Statistical characteristics of the PC properties of various compositions.

Source of Variability	SS	DF	MS	F-Stat	*p*-Value	Conclusion
**Density**						
Between samples	4.67	2	2.33	0.17	0.842	Insignificant
Between compositions	11,393.33	6	1898.89			
Residual (error)	160.67	12	13.39			
Total	11,558.67	20				
**Compressive strength**						
Between samples	7.49	2	3.74	18.02	0.001	Significant
Between compositions	251.91	6	41.99			
Residual (error)	2.49	12	0.21			
Total	261.89	20	–			
**Water absorption**						
Between samples	0.01	2	0.00	3.12	0.081	Insignificant
Between compositions	0.53	6	0.09			
Residual (error)	0.01	12	0.00			
Total	0.55	20				
**Coefficient of thermal conductivity**						
Between samples	0.00	4	0.00	0.77	0.553	Insignificant
Between compositions	0.09	6	0.01			
Residual (error)	0.00	24	0.00			
Total	0.09	34	–			

Sum of Squares—SS. Degrees of Freedom—DF. Mean Square—MS.

**Table 7:** Effect of Various Types of Fillers and Aggregates on the Properties of Polymer Composites.

Ref.	Component	Results Obtained
[39]	Perlite fine aggregate	The inclusion of 5–10% expanded perlite in polymer concrete does not significantly reduce its durability and ensures properties comparable to those of control mixtures.
[64]	Recycled glass aggregate made from cathode ray tube glass waste	RS with a low content of finely dispersed recycled glass aggregate (up to 10%) are lightweight and have good chemical, physical, and mechanical properties. The optimal combination of recycled glass aggregate and sand ensures dense particle packing.
[65]	Recycled aggregate from crushed concrete aggregate	RS with recycled aggregate have a higher compressive strength of 96 MPa than RS with natural aggregate (89.1 MPa).
[66]	Fly Ash Filler	A combination of 30% resin and 70% filler produces a polymer composite with a compressive strength of 66.19 MPa at 3 days of age and a density of 1.88 g/cm^3^.
[67]	Waste Rubber Powder	The strength of the polymer composite decreases by 22.4% with the inclusion of 7.5% waste rubber powder. Composite shrinkage is also reduced.
[68]	Palm Oil Ash Microfiller	Palm oil fuel ash provides excellent filling capacity and high compressive strength for polymer concrete.
[69]	Lightweight Ceramic Waste Fillers	Using a ceramic filler with a grain size of 4–8 mm with an epoxy binder allows for polymer concrete with a compressive strength of 99.68 MPa and a flexural strength of 18.71 MPa.
[70]	Fly Ash	Including 10–20% fly ash filler by weight improves the chemical resistance and strength properties of the polymer composite.
[71]	Quartz flour	The hardness and flexural modulus of a polymer composite made from unsaturated polyester resin increase with the addition of quartz flour up to 40% by weight.

## Data Availability

The original contributions presented in the study are included in the article, further inquiries can be directed to the corresponding author.

## Abbreviations

The following abbreviations are used in this manuscript:

GGBFS	Ground granulated blast-furnace slag
MC	Micro-calcite
QF	Quartz flour
PC	Polymer concrete
